# A new benchmark illustrates that integration of geometric constraints inferred from enzyme reaction chemistry can increase enzyme active site modeling accuracy

**DOI:** 10.1371/journal.pone.0214126

**Published:** 2019-04-04

**Authors:** Steve J. Bertolani, Justin B. Siegel

**Affiliations:** 1 Department of Chemistry, University of California Davis, Davis, California, United States of America; 2 Department of Biochemistry and Molecular Medicine, University of California, Davis, Davis, California, United States of America; 3 Genome Center, University of California Davis, Davis, California, United States of America; Weizmann Institute of Science, ISRAEL

## Abstract

Enzymes play a critical role in a wide array of industrial, medical, and research applications and with the recent explosion of genomic sequencing, we now have sequences for millions of enzymes for which there is no known structure. In order to utilize modern computational design tools for constructing inhibitors or engineering novel catalysts, the ability to accurately model enzymes is critical. A popular approach for modeling enzymes are comparative modeling techniques which can often accurately predict the global structural features. However, achieving atomic accuracy of an active site remains a challenge and is an issue when trying to utilize the molecular details for designing inhibitors or enhanced catalysts. Here we explore integrating knowledge about the required geometric orientation of conserved catalytic residues into the comparative modeling process in order to improve modeling accuracy. In order to investigate the utility of adding this information, we first carefully construct a benchmark set of reference structures to use. Consistent with previous findings, our benchmark demonstrates that the geometry between catalytic residues across an enzyme family is conserved and does not tend to deviate by more than 0.5Å. We then find that by integrating these geometric constraints during modeling, we can double the number of atomic level accuracy models (<1Å RMSD to the crystal structure ligand) within our benchmarking dataset, even for targets with templates as low as 20-30% sequence identity. Catalytic residues within an enzyme family are highly conserved and can often be readily identified through comparative sequence analysis to a known structure within the enzyme family. Therefore utilizing this readily available information has the potential to significantly improve drug design and enzyme engineering efforts for which there is no known structure for the enzyme of interest.

## Introduction

The atomic structure of an enzyme is crucial in the design of novel therapeutics [1], understanding of function [2], and our ability to re-engineer their functionality [3]. Therefore, many excellent *in silico*-based methods have been developed to predict an enzyme’s structures from the protein sequence. One of the most commonly used techniques is homology modeling, where the central tenet is that the structure a sequence will fold into is primarily dictated by the degree of sequence homology that the query sequence has to the solved crystal structure (template). It is generally accepted that the closer in sequence identity of the query and the template(s), the more accurate the modeling will be [2]. While the proliferation of homology modeling tools has resulted in the ability to generate molecular models in which the general fold and placement of amino acids can be accurately predicted for roughly 70% of protein sequences [4], a 0.5Å error in the backbone can lead to 15% decrease in accuracy of the *χ*_1_ angle when modeling side chains [5]. These errors in the placement of side chain atoms can limit the model’s usefulness when either designing therapeutics or carrying out molecular analysis to understand and re-engineer protein function.

One of the most promising approaches in recent years for improving the atomic accuracy in modeling is through the integration of readily attainable sparse experimental or bioinformatics data. For example, when deuterated NMR NOE constraints are added to model building, models generally gained over a 1Å increase in accuracy [6]. In other work, low resolution cryo EM maps have been added to enable solutions for eight out of thirteen X-ray datasets that were not solvable with any other technique [7]. To build on this growing realization that integrating data can be used to enhance modeling accuracy, we hypothesized that the integration of a new type of readily available data could be used when modeling enzymes: the mechanistic data of enzyme reactions that has been obtained through enzymology studies over the last several decades. It is well established that the precise geometric orientation of catalytic residues within an enzyme is critical for the enzyme to catalyze the chemical reaction [8][9][10]. Therefore, each catalytic residue that participates in the reaction must have a precise location relative to all of the other catalytic residues. In fact, this conservation of the spatial orientation has been known for some time and previously studied in depth by the Thornton group [9]. Here, we propose that adding geometric constraints that enforce the catalytic residues to maintain a catalytically viable arrangement for the enzyme’s reaction during modeling will result in a general increase in modeling accuracy, particularly within the active site.

**Fig 1:**
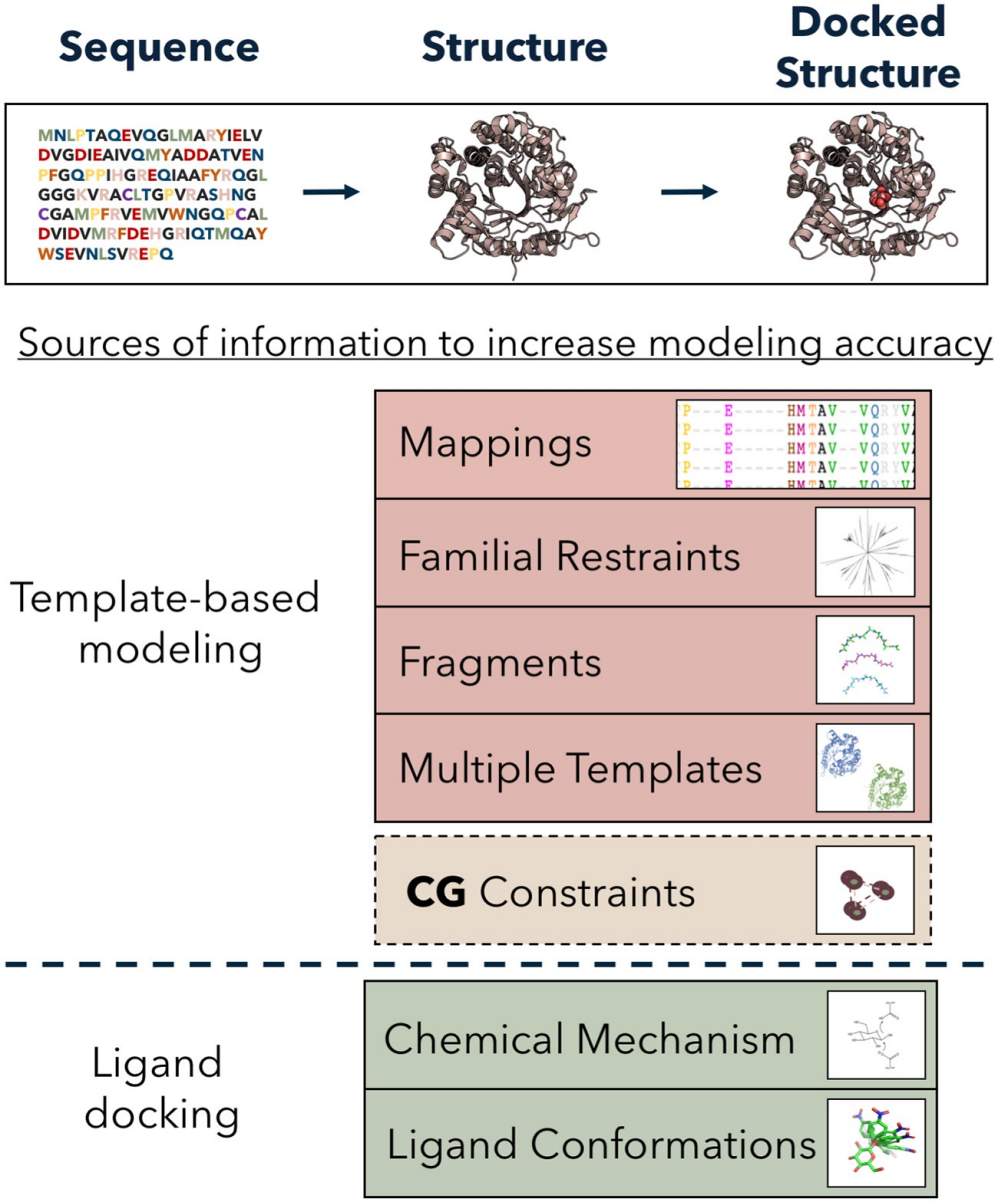
Addition of information to augment template-based modeling and ligand docking protocols. **Sequence** → **Structure** Beginning with a target sequence, *apo* protein structures can be predicted. The accuracy of these models has been shown to increase with the addition of other sources of information, including accurate sequence-structure mappings (alignments) [13], familial (evolutionary) constraints [14], multiple templates [15] and fragments [16] **Structure** → **Docked Structure** Taking the *apo* structure and docking a ligand into results in a docked structure, with a ligand bound. The accuracy of these models has been shown to increase with the addition of ligand conformational degrees of freedom and chemical mechanism constraints. By adding **CG** constraints, we measure the increase in accuracy over the entire pipeline.

In the effort to measure the expected increase in accuracy by integrating knowledge of catalytic geometry, we constructed a benchmark set of target PDB structures and follow a combination of homology modeling protocols with subsequent docking of ligands as inspired by recent landmark studies [11]. Here we find, over a subset of crystal structures in the same enzyme family, the distances between pairs of *C*_*α*_ and *C*_*β*_ atoms on catalytic residues deviate by no more than 0.5Å, from the catalytically viable arrangement, a result consistent with previous work on this topic [12]. We demonstrate that incorporating this knowledge, in the form of distance constraints, when modeling enzymes results in an increase in modeling accuracy of the enzyme active site. We see an improvement in the placement of the catalytic residue *C*_*α*_ atoms by an average of 0.3Å We also show that an improvement in the accuracy of the model leads to improved accuracy in important downstream applications such as docking. Using catalytic geometry (**CG**) constraints as an augmentation to standard homology modeling methods (Fig 1), we observe the number of ligands docked into models achieving atomic accuracy (i.e. <1Å RMSD) is doubled relative to current state of the art modeling protocols. This improvement is observed for modeling problems both when the query sequence is close and distal in sequence homology to the template(s).

## Results

### Verifying conservation of inter-catalytic residue geometries

**Fig 2:**
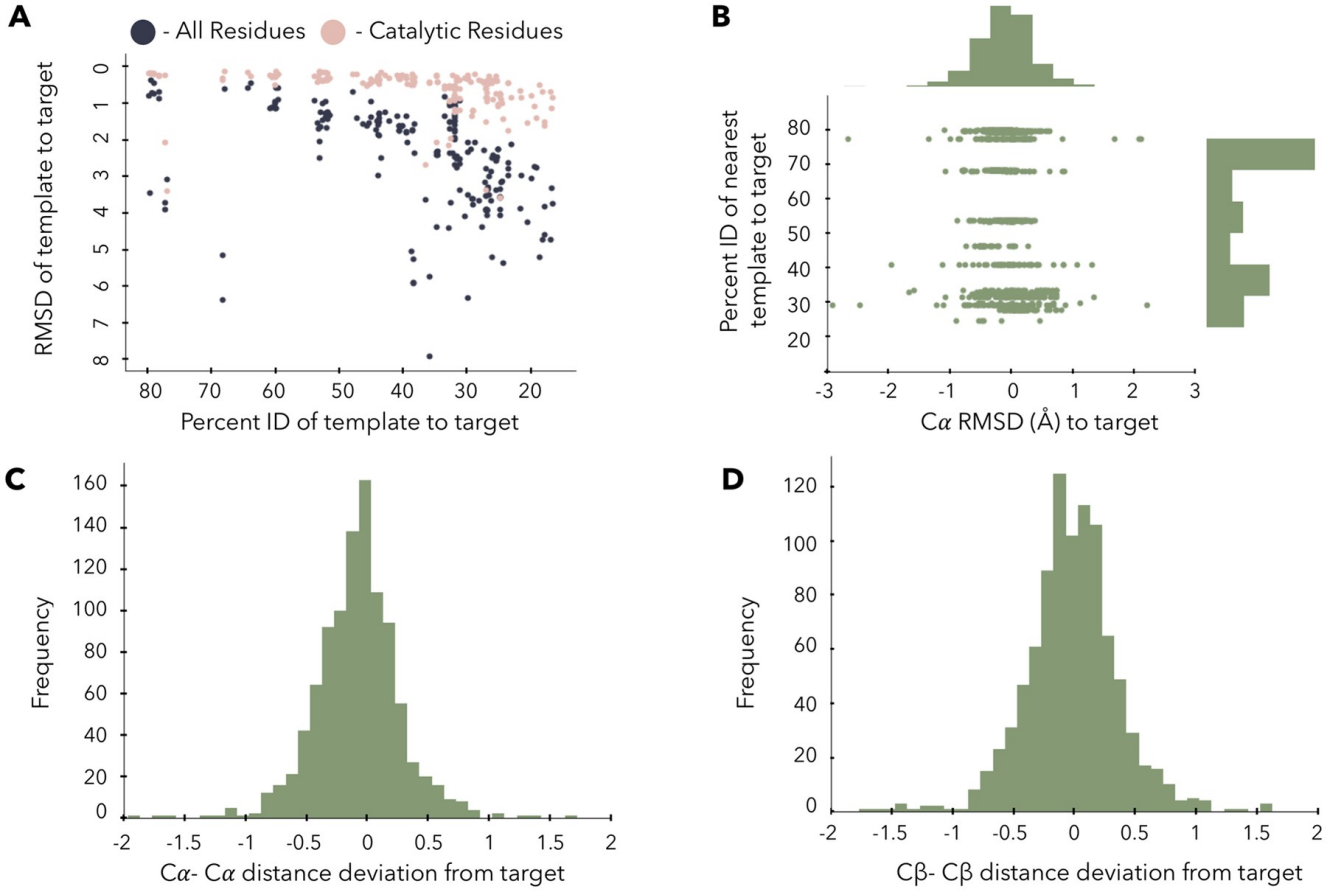
Structural informatics of conservation of inter-catalytic residue atom distances **A**- The RMSD of each target to each template structure is shown. The RMSD over just the catalytic residues (light pink), and the overall protein RMSD values are shown as a function of the sequence homology of each template. **B**—Distribution of the benchmark template percent identity to target sequences and the deviation of the *C*_*α*_ of each template to the target catalytic residues. **C**- Deviation of *C*_*α*_—*C*_*α*_ distances to the reference distances in the target crystal structure. Note the gaussian shape with a 0.5Å deviation. **D**- The *C*_*β*_—*C*_*β*_ deviation to reference measurements, again with a narrow distribution. The distance deviation distribution of *C*_*α*_-*C*_*β*_ are similar to that of *C*_*β*_-*C*_*β*_ and are centered at 0 with an approximate deviation of 0.5Å (S1 Fig).

We demonstrate in our benchmark that the distances between corresponding pairs of catalytic residue atoms are conserved for enzymes within a family performing the same type of chemical reaction (i.e. all trypsin-like serine proteases cleave a peptide carbonyl bond using a serine that is backed up by a histidine-aspartate network [17]). Each target within our benchmark set was carefully selected to ensure that catalytic residues were in a conformation consistent with the reaction mechanism. To measure the distance variability between conserved catalytic residues, we structurally overlaid up to ten homologs that ranged from 20%-80% identity onto the structure of the query sequence and calculated the *C*_*α*_ RMSD over both the entire protein and over just the catalytic residues. As sequence homology decreases so does structural homology (Fig 2A, dark purple points), consistent with the prior work in the field [18][19]. However, the catalytic residues maintain within 1Å *C*_*α*_ RMSD (Fig 2A, light pink points), even as the sequence homology decreases down to the <30% levels. These results are consistent with known literature [12].

To estimate the allowed distance deviation of catalytic residues for modeling, we systematically measure the distances between each pair of atoms on the target catalytic residues (*C*_*α*_—*C*_*α*_, *C*_*α*_—*C*_*β*_, *C*_*β*_—*C*_*β*_), and then the equivalent pair on the template and calculate the difference. We find that for both the *C*_*α*_—*C*_*α*_ and the *C*_*β*_—*C*_*β*_ distances, the deviation does not vary much as the sequence homology varies (Fig 2B). The deviation from the catalytically viable arrangement is near 0.5Å for both the *C*_*α*_—*C*_*α*_ and *C*_*β*_—*C*_*β*_ atom to atom distances for catalytic residues. The deviation histograms (Fig 2C and 2D) are normally distributed around 0 with a standard deviation of roughly 0.5Å. These results agree with the approximately 0.5-0.6Å deviation previously seen by the Thornton group [12].

### Model improvement

Given this observation of stringent structural conservation across sequence space for enzymes that catalyze related reactions, we explored adding these distances as constraints to the system. For each target in the benchmark, homology models were made with and without adding **CG** constraints. In order to add **CG** constraints into RosettaCM [20], the distances between each pair of catalytic residues atoms (both *C*_*α*_ and *C*_*β*_ atoms) are set using a harmonic restraint. For example, to constrain the distance between the *C*_*β*_ atoms of catalytic residues CYS-73 and CYS-217, the following constraint is added to the simulation:

AtomPairCB73CB217SCALARWEIGHTEDFUNC1000HARMONIC8.230.5

**Fig 3:**
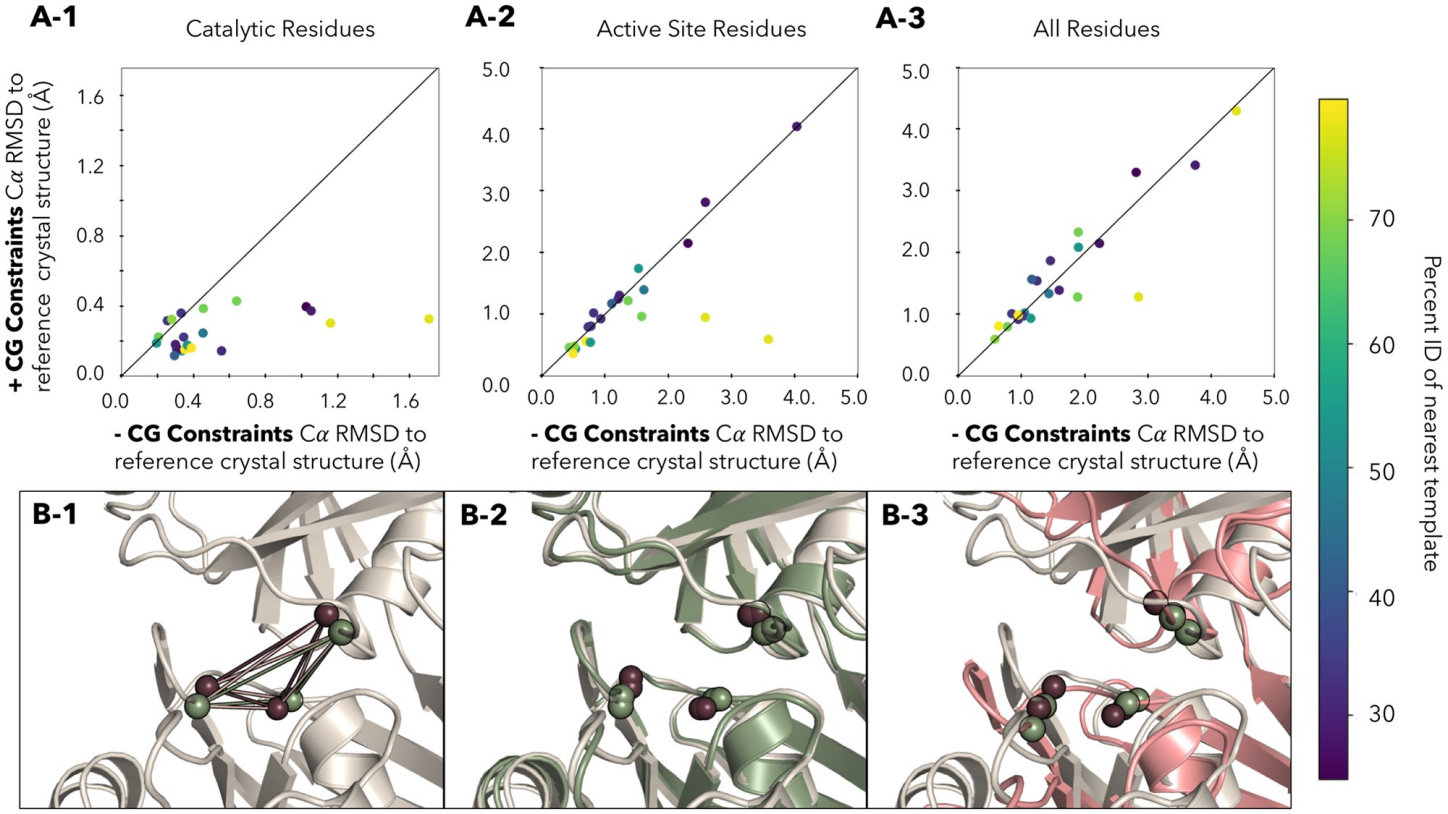
Adding CG constraints to *apo* models **A-1** Catalytic Residues **A-2** Active Site Residues (8Å), **A-3** All Residues—The Y axis contains the *C*_*α*_ RMSD to the reference crystal structure with the **CG** constraints added. The X axis contains the *C*_*α*_ RMSD of to the reference crystal structure of the standard homology modeling. The RMSD is calculated for different sets of residues, over either just the catalytic residues, the residues predicted to be in the active site, or over the entire protein. Points below the line indicate that including the distances helps the model accuracy. Points on the line demonstrate that there was no improvement in accuracy. Targets are colored by the difficulty based on sequence homology of the closest template used for modeling. Bottom—In each cell, the *C*_*α*_ atoms of each catalytic residues (Cys-73, Cys-217 and Glu-208) are shown in burgundy. Each of the *C*_*β*_ atoms are shown in green. **B-1** Crystal structure of benchmark target *2gke* (grey). Lines drawn between indicate the measurements between atoms that are used as **CG** constraints. **B-2**—With the addition of the **CG** constraints to the model, the catalytic residues of the **CG** constrained model (green) overlays with the crystal (grey). **B-3**—Standard homology model (red) with the *C*_*α*_ and *C*_*β*_ atoms shown as spheres. All results in this figure are from the best of the five lowest in energy models, the equivalent figure illustrating results from the single lowest energy model with addition of **CG** constraints is presented in S2 Fig.

This sets that distance to be 8.23Å +/- 0.5Å and the constraint is upweighted by the factor of 1000. This factor ensures that the **CG** constraints have a higher weight than any other restraints on the system. In the modeling accuracy analysis we evaluated the minimum RMSD value from a pool of the lowest 5 models based on energy (Fig 3A1, 3A2 and 3A3), as well as the single lowest energy model (S2 Fig). In each case, the RMSD between the model and the reference crystal structure can be measured between the *C*_*α*_ atoms in a) the catalytic residues b) the active site residues c) the entire protein structure. With the addition of **CG** constraints, the model accuracy increases by 0.3Å on average for the catalytic residues (Fig 3).

This indicates that the catalytic residues are modeled as expected from the constraints. As more residues are included in the analysis (active site) the improvement decreases to 0.2Å, and over the whole protein, the improvement is lost. This indicates that the **CG** constraints enforce the proper placement of the catalytic residues, but in general don’t act as lynchpins that improve modeling accuracy beyond the proper placement of the catalytic residues. The data follows the general trend that the closer the template is to the target sequence, the more accurate the active site *C*_*α*_ atom placement is (models built from templates closer in sequence identity are closer to the origin—Fig 3A-1, 3A-2 and 3A-3).

In one particular case, 2gke, we observed that enforcing correct placement of the *C*_*α*_ and *C*_*β*_ atoms of catalytic residues through **CG** constraints was crucial for accurate modeling of enzymes. This was particularly noteworthy as 2gke has high homology (79%) to the template used and would normally be considered an “easy” homology modeling problem. However, when modeled with without **CG** constraints the catalytic residues RMSD go from 1.8Å *C*_*α*_ RMSD to 0.4Å *C*_*α*_ RMSD. In addition, the overall structure goes from 2.8Å to 0.9Å *C*_*α*_ RMSD.

### Ligand docking improvement

Given the improvement in the accuracy of the placement of the catalytic residues, we expected that we would see improvements in the accuracy of downstream applications like docking. We docked the conformational ensembles of the known ligand into the five lowest energy models from the modeling step, allowing different structures as starting points for the same target, and enforced the ligands to dock in a manner consistent with the enzyme mechanism [21].

The models used to evaluate effectiveness of CG constraints were passed through four filters. First, the lowest 10% of structures based on overall protein system score were chosen from each of the five starting models and combined into a pool of structures. Second, the pool of structures were filtered to remove any models with a single enzyme constraint greater than one, indicating the model was inconsistent with known mechanistic chemistry. Third, the lowest 50% based on active site energy were kept, summing energies from the residues predicted to be within 8Å of the active site. Fourth, the models were sorted based on the ligand interface score. Finally, out of the 5 structures with the lowest interface score, we select a) the lowest interface energy model (S3 Fig) and we select b) the lowest RMSD model from those 5 structures. In the first analysis (a) we are testing how well we perform if we trust the accuracy of the energy function to discriminate between native and non-native poses. In the second analysis, we analyse our best 5 models based on energy, with the understanding that the energy function may not be able to distinguish between the most native like pose and other structures similar in energy. This is an approach commonly used in CASP-style competitions [22], were multiple submissions for structure predictions are allowed.

**Fig 4:**
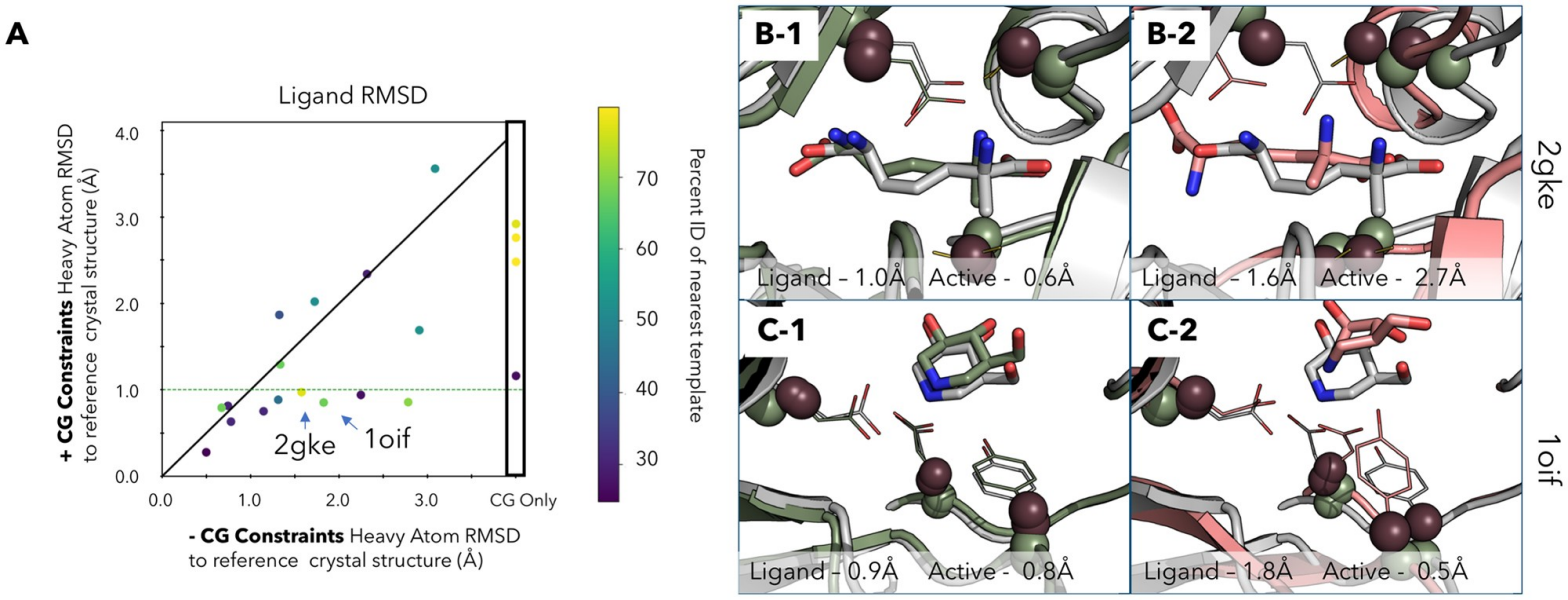
Docking with and without CG constraints. **A** Ligand RMSD over all heavy atoms in the ligand versus the reference crystal structure ligand. The y-axis are RMSD of the **CG** constraint added models, the x-axis are RMSD of the standard models. Points below the diagonal are improved with the addition of **CG** constraints. Points on the line are not affected and points above the line are worse with the addition of **CG** constraints. The green line marks the 1Å RMSD cutoff for the models made with **CG** constraints. On the x-axis there are 4 targets that were unable to find a solution without the addition of **CG** constraints (CG only). Targets are colored by the difficulty based on sequence homology of the closest template used for modeling. 2 targets are not shown as they were unable to identify solutions in either case. **B-1** Docking results for target 2gke with CG constraints, crystal structure (grey) overlaid with model (green). **B-2** Docking results for target 2gke without CG constraints, crystal structure (grey) overlaid with model (red). **C-1** Docking results for target 1oif with CG constraints, crystal structure (grey) overlaid with model (green). **C-2** Docking results for target 1oif without CG constraints, crystal structure (grey) overlaid with model (red).

We find 16 targets in which both standard modeling and **CG** modeling identify structures that are consistent with the mechanistic detail known for the system. When **CG** constraints were not added, only 4/16 of those ligands were placed within a 1Å RMSD to the crystal structure, whereas with the inclusion of the **CG** constraints, 10/16 sub 1Å RMSD models were identified (Fig 4). This is a 2.5-fold increase in the number of sub 1Å accuracy models that can be modeled by including the **CG** constraints.

In an ideal case (Fig 4—2gke) the addition of a several select distances greatly impacts the accuracy of the ligand and the enzyme structure. Here, several catalytic residues are located on loops. With standard homology modeling techniques, the ligand heavy atom RMSD is 1.6Å from the reference crystal structure ligand. The flexibility of loops has been long known in the literature as problematic for structure prediction [23] and it is no surprise that the loop is not correctly placed for the chemical reaction to take place. With the definition of catalytic residues, the cysteines are correctly placed to accommodate the reaction yielding a ligand heavy atom RMSD of 1.0Å. The impact in this case is an improvement of 0.6Å in the placement of the ligand, which can be seen to recover nearly all the correct contacts at the interface (S1 PDF).

In another case (Fig 4—1oif) the addition of several distances between catalytic residue *C*_*α*_ and *C*_*β*_ atoms improves ligand RMSD by 0.9Å. Interestingly, in this case the addition of the **CG** constraints increases the active site RMSD by 0.3Å (although it is still sub 1Å). This example demonstrate how small details in the orientation of catalytic residues can have a large impact on the docking results, and that RMSD-like metrics can be a misleading metric for evaluating the actual goal of accurately predicted active site-ligand molecular interactions.

We also find that for 20% of targets (4/20) the only way to get a viable structure was by including the **CG** constraints. In these cases, the models without the **CG** constraints applied during modeling, were unable to find a docked model that also satisfied the enzyme mechanism constraints. For example, 1xpz was unable to identify a low energy model that bound the metal in a catalytically viable arrangement and therefore no standard models were identified as being consistent with reaction mechanism knowledge (S1 PDF 5.17-5.20). However, the models with **CG** constraints were accurate enough that solutions were able to be found. There are 2 targets which find no models. In both cases (1eh5, 1tqh) the nearest template identified were 24 and 31% similar (S1 PDF 5.21-5.22). These targets are both in the difficult category in which homology modeling is known to have failures.

## Discussion

As many other recent techniques that improve homology modeling accuracy have found, including sparse experimental data can lead to drastic improvements in model accuracy. We proposed that the decades of research studying enzyme reaction mechanisms can be an additional source of sparse data that can improve modeling of enzymes. Specifically, catalytic residues of enzymes are known to adopt a specific geometry as they catalyze chemical reactions. However, typically this knowledge about how the chemical reaction takes place is not used during homology modeling of enzymes. Here we create a benchmark to demonstrate that sparse constraints that describe the arrangement of catalytic residues in the enzyme can be included to increase model accuracy. The increase in accuracy is seen especially in the placement of the *C*_*α*_ atoms of the catalytic residues, where an average improvement of 0.3Å is seen over standard methods. This can have a drastic effect on the accuracy of ligand docking, as we have shown this method yields 2-fold more structures with ligands docked below 1Å RMSD accuracy.

Using our benchmark, we demonstrate that the distances between pairs of *C*_*α*_ and *C*_*β*_ atoms on catalytic residues are conserved and can be used in general as a constraint. This indicates that catalytic residues in enzymes are typically prearranged to perform catalysis in both the position and the angle in which the side chains of the catalytic residues are pointing. Therefore, these distances could be measured from a single solved homologous structure, as long as it is in a catalytically viable arrangement, and the distances could be applied to modeling hundreds or thousands of related target sequences.

Another interesting result is how few catalytic residues must be defined to get an improvement. In the case of 2gke, a diaminopimelate epimerase, the addition of **CG** constraints between 3 catalytic residues allows for recovery of all of the atomic contacts in the docked structure (Fig 4—2gke) with a heavy atom ligand RMSD of 1.0Å. In contrast, with the standard modeling methods the ligand has a higher RMSD by 0.6Å and upon analysis of the active site few if any of the molecular interactions between the protein and ligand are recovered. This demonstrates that the inclusion of **CG** constraints, which can improve the accuracy of the model, can lead to improvements in accuracy for downstream applications like ligand docking. This holds a lot of potential for new methods which require the modeling of an enzyme active site for either the accurate docking of new drugs or modeling enzymes in genomic mining efforts to discover new function ([24] and [25]).

While integration of **CG** constraints can increase modeling accuracy, one drawback of this technique is that it requires detailed knowledge and understanding of the reaction mechanism. There are thousands of enzymes for which mechanistic studies have been conducted, but for enzymes where the reaction mechanism is unclear, this method will not be of use. This technique also requires the identification of a homologous enzyme for which there is a known structure in a catalytically relevant conformation from which to measure the catalytic residue **CG** constraint distances. Fortunately, there are structures in many different classes of enzymes that are suitable as well as a large number of theoretical enzyme active site studies which may also provide inter-catalytic residue distance information. Given that our benchmark is comprised of monomeric enzymes, we have yet to explore how integration of this type of data will affect modeling accuracy in enzymes where the active sites are composed of multiple symmetric subunits.

Overall, we have demonstrated the stringent structural conservation of catalytic residues within enzyme active sites can be utilized for improving protein modeling accuracy. This adds to a growing body of knowledge that integration of previously established or readily obtainable experimental data into protein modeling is an effective approach of generating atomically accurate molecular models. Due to the exponentially growing sequence databases the importance of being able to computationally generate atomically accurate model of a protein is becoming of paramount importance, and methods such as these are likely to play an essential role in the future of biomolecular studies.

## Methods and materials

### Construction of benchmarking set of PDB crystal structures

In order to evaluate if addition of **CG** constraints increases accuracy, a set of target PDB crystal structures was selected to predict using homology modeling and ligand docking techniques. For a crystal structure to have residues that are in the catalytically viable arrangement, we restricted our PDB search to only structures that had either a transition-state inhibitor, substrate, reactant or product bound to the active site. In addition, the transition-state, inhibitor, substrate or product was required to be bound in a catalytically productive and relevant orientation based on the current understanding of the enzymes mechanism. All of these protein structures are monomeric and cover a range of protein lengths, from 131 to 583 amino acids. They cover the EC classes 3,4 and 5, a range in the number of catalytic residues, from 2 up to 6, and a range of difficulties in homology modeling, from easy (>60%) to hard (<30%) (Table 1).

**Table 1:** The benchmark set of enzymes with their catalytic residues in a catalytically viable arrangement. This set covers EC classes 3,4,5, a range of lengths, difficulties and mechanisms These are all monomeric enzymes with a ligand bound in the active site. The approximate difficulty of the modeling target is shown in percent identity (PID) column, which gives the sequence homology of the closest template used in modeling.

PDB Code	Length of Protein Sequence	Catalytic Residues	EC	Detail	PID(%) Nearest Templates	Reference
1ogx	131	16,40,103	5.3.3.1	Ketosteroid isomerase	32.8	[28]
1oh0	131	16,40,103	5.3.3.1	Ketosteroid isomerase	33.6	[29]
1w6y	131	16,40,103	5.3.3.1	Ketosteroid isomerase	33.6	[30]
1p6o	161	62,64,91,94	3.5.4.1	Cytosine deaminase	27.7	[31]
4fua	215	73,92,94,155	4.1.2.17	L-fuculose-1-phosphonate aldolase	40.9	[32]
2nlr	234	104,120	3.2.1.4	Endoglucanase	70.7	[33]
1tqh	247	25,94,193,223	3.1.1.1	Carboxylesterase	31.6	[34]
1ney	247	12,95,165	5.3.1.1	Triosephosphate isomerase	53.4	[35]
1xpz	258	94,96,119,199	4.2.1.1	Human carbonic anhydrase	79.8	[36]
1jcl	260	47,102,137,167,201	4.1.2.4	Deoxyribose-phosphate aldolase	32.3	[37]
3ia2	271	28,94,95,222,251	3.1.1.2	Esterase	53.9	[38]
2gke	274	73,208,217	5.1.1.7	Diaminopimelate epimerase	77.4	[39]
1eh5	279	41,115,233,289	3.1.2.22	Palmitoyl-protein thioesterase 1	24.7	[40]
2jaj	289	78,172,268,273	3.5.3.18	Dimethylarginine dimethylaminohydrolase 1	29.9	[41]
1h2j	303	139,202,228	3.2.1.4	Endoglucanase	68.0	[42]
6cpa	307	69,72,145,196,248,270	3.4.17.1	Carboxypeptidase A	79.5	[43]
1hqd	320	17,87,88,264,286	3.1.1.3	Lipase	77.3	[44]
3veu	386	32,219	3.4.23.46	Human beta secretase	53.3	[45]
2jie	454	167,298,356	3.2.1.21	*β*-glucosidase B	46.4	[46]
1oif	468	166,295,351	3.2.1.21	Family 1 *β*-glucosidase	68.3	[47]
1oim	468	166,295,351	3.2.1.21	Family 1 *β*-glucosidase	68.3	[47]
1ju3	583	44,117,118,259,287	3.1.1.84	Cocaine esterase	29.3	[48]

### Template identification and alignments

In order to identify templates to be used for homology modeling, each target sequence was searched using HMMER3 [26] against the PDB database. The full protein sequences were downloaded for all significant matches, then these sequences were aligned to the target sequence using PROMALS3D [27], and any match with over 80% similar to the target sequence was removed. This removes the best matching structures for modeling, and ensures that the models will be built without bias towards the answer. Up to 10 templates below the 80% threshold were kept for modeling. In addition, to increase the sampling efficiency of the modeling, each target sequence was trimmed, by removing from the N & C termini any portion of the sequence that had no coverage by any template. The templates identified and used for each modeling target are attached (S4 Fig).

### Definition of catalytic residues and enzyme mechanism chemistry

For each target, the literature was searched to identify the catalytic residues. In many cases, these follow from well-established mechanistic studies. In addition, detailed studies of each mechanism were encoded in the form of an enzyme constraint file [21], which specifies precisely how an enzyme interacts with the substrate according to the reaction chemistry. These have previously been used to ensure that the ligands are docked in a way that is consistent with mechanistic knowledge. For each of these constraint files, because the crystal structures were restricted to those with ligands interacting in a way consistent with how the reaction mechanism takes place we are able to create the enzyme design constraint files using common physical chemistry knowledge (i.e. using ideal bond distances and angles). The enzyme mechanism docking constraints were verified by recovering the correct conformation in the crystal structure target (S1 PDF).

### Spatial conservation of the *C*_*α*_ and *C*_*β*_ atoms of the catalytic residues for enzymes

Each target PDB structure and the templates identified create a set of proteins in the same enzyme family. Given how the targets are selected for the benchmark, the catalytic residues are in a catalytically viable arrangement. The templates have no such selection criteria, and are only required to have sequence homology to the target sequence. Therefore, by overlaying the templates onto the target PDB, and measuring the distance between 2 atoms on separate catalytic residues (*C*_*α*_ and *C*_*β*_ atoms) and repeating that measurement for many solved crystal structures in the same enzyme family, an estimate of the allowed deviation between those 2 atoms can be calculated.

For a given target sequence ***S***, with catalytic residues ***A*** and ***B***, the Euclidean distance *d* can be measured d = m(**A**,**B**) between specific atoms. The equivalent distance of equivalent pairs of catalytic residues **A’** and **B’** on template structure ***T*** can be measured d’ = m(**A’**,**B’**). Here, the equivalent distance can be identified using the sequence alignments which map sequence ***S*** to sequence ***T***. The difference (Δ = d − d’) between the target distance and the template distance approximates the deviation from the catalytically viable arrangement found in the templates. By enumerating all combinations of catalytic residues over *C*_*α*_ and *C*_*β*_ and then measuring the equivalent pairs of catalytic residues in the template structures, and plotting the difference, an estimate of the naturally occurring deviation from the catalytically viable geometry can be calculated.

### Calculation of CG constraints

We restrict out measurements to the distances between *C*_*α*_ and *C*_*β*_ atoms. For the simulations performed, the distances between the *C*_*α*_—*C*_*β*_, *C*_*β*_—*C*_*β*_ and *C*_*α*_—*C*_*β*_ atoms were measured on the target crystal structure and implemented as harmonic distances constraints in Rosetta with a 0.5Å tolerance (S2 PDF for example). This assumes that these distances may be measured or calculated a priori within an error of 0.5Å.

### Homology modeling

For each target sequence, custom fragments files were created using ROBETTA [49][50] in benchmarking mode which removes any fragments within 80% of the target sequence to remove biasing the models toward the correct solution. Using the aligned sequences of the target and the templates identified, evolutionary constraints were calculated and used for modeling [14]. Evolutionary constraints are previously identified residue to residue distance constraints which supplement the Rosetta score function by giving a bonus to satisfying each constraint found. The exact same modeling protocol was used for homology modeling and docking, with the single change of adding of *C*_*α*_—*C*_*β*_, *C*_*β*_—*C*_*β*_ and *C*_*α*_—*C*_*β*_ constraints (Fig 1). For each target sequence, 100 models were generated using RosettaCM [20], and either the single lowest energy model or the lowest five models were selected for docking (five structure submissions are used for other protein structure prediction assessments [51][22]). The structures selected were chosen by summing residue energies for residues predicted to be in the active site based on bioinformatics and sorting on that energy.

### Docking

The target ligands were prepared by taking the ligand from the crystal structure of the target. Each ligand was converted in Spartan16 [52] to complete bond valences (or lack thereof for substrates that were covalently bound to the enzyme structure). The degrees of freedom were frozen for atoms and bonds connected to those atoms that participate in the chemical reaction with the enzyme. All other torsional angles were sampled using the PM3 semi-empirical forcefield [53]. The lowest 100 energy conformations were kept for use in docking. All ligands were parameterized for use in RosettaDock [54][55] including atomic charges from the Spartan16 ‘Electrostatic’ option.

Each docking simulation started by placing the ligand at the average position between all of the catalytic residues defined for that target. This was followed by 3 iterations of perturbing the ligand in a 20Å grid, optimizing the catalytic constraints, and sampling/minimizing the enzyme side chains and ligand conformations.

For each of the 5 lowest energy *apo* homology models, 100 simulations were ran, which resulted in 500 model structures. The following four filters were used to select the final models for analysis. First, for each of the lowest energy homology models, the lowest 10% based on overall protein score (total_score) were selected and combined into a pool. Second, from the pool, any structure which had a single enzyme constraint greater than one Rosetta Energy Unit was removed. Third, the lowest 50% based on the predicted active site energy (summing the residues predicted to be within 8Å of the active site) were kept. Fourth, the models were sorted based on ligand interface score. Either the lowest interface energy model post-filtering (S3 Fig) or lowest ligand RMSD models (Fig 4A) from the lowest five models based on interface energy were selected for analysis.

### Data availability

The benchmark files can be found at https://www.github.com/sjbertolani/benchmark-lite and the files are described in S3 PDF as well as in README files throughout the linked data repository.

The following version of Rosetta was used to perform the work: 17be250fab3b65d60d806025d7219a5373754924.

## Supporting information

S1 Fig Informatics additional data. *C*_*α*_—*C*_*β*_ Distribution of distances from benchmark crystal structures.
(PNG)

S2 Fig *Apo* lowest single energy structure. Results for apo modeling of protein sequences by selecting the lowest *single* structure based on energy. See S1 PDF—5.11 for further discussion of the point located at (2,7) on the Active Site Residues plot. This is an artifact of a terminus flipped in versus out in the models.
(PNG)

S3 Fig Docked lowest single energy structure. Results for docking by selecting the lowest *single* structure based on energy. One extreme point is not shown.
(PNG)

S4 Fig Templates used for modeling. (PNG)

S1 PDF Benchmark docking results. (PDF)

S2 PDF Example of harmonic distance constraints. (PDF)

S3 PDF Modeling details and description of files. (PDF)

S1 Files Benchmark files. (GZ)
