# Supplementary figures and images for: A new benchmark illustrates that integration of geometric constraints inferred from enzyme reaction chemistry can increase enzyme active site modeling accuracy
Source: PLoS One. 2019 Apr 4;14(4):e0214126. doi: 10.1371/journal.pone.0214126 (PMC6448891; doi:10.1371/journal.pone.0214126)

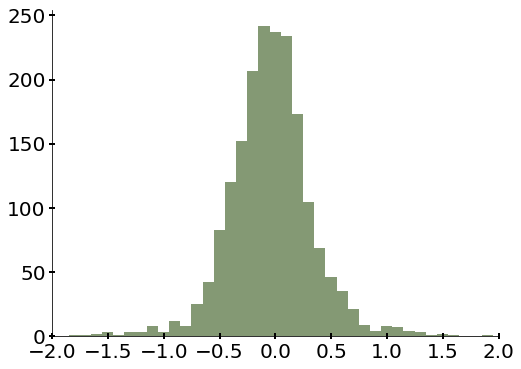

Supplement: S1 Fig — Cα—Cβ Distribution of distances from benchmark crystal structures. (PNG) [file pone.0214126.s001.png]

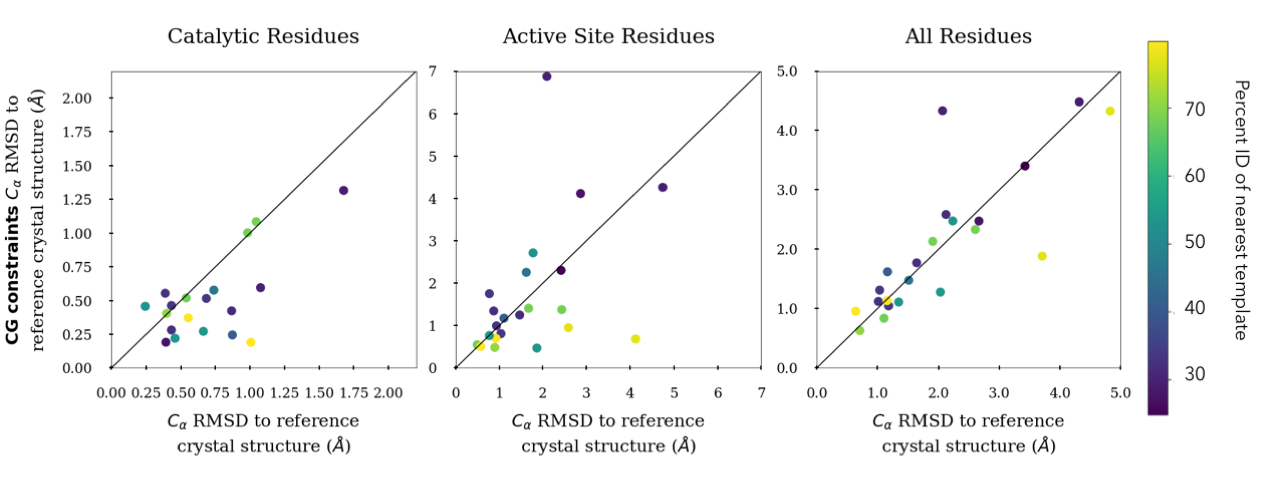

Supplement: S2 Fig — Results for apo modeling of protein sequences by selecting the lowest single structure based on energy. See S1 PDF—5.11 for further discussion of the point located at (2,7) on the Active Site Residues plot. This is an artifact of a terminus flipped in versus out in the models. (PNG) [file pone.0214126.s002.png]

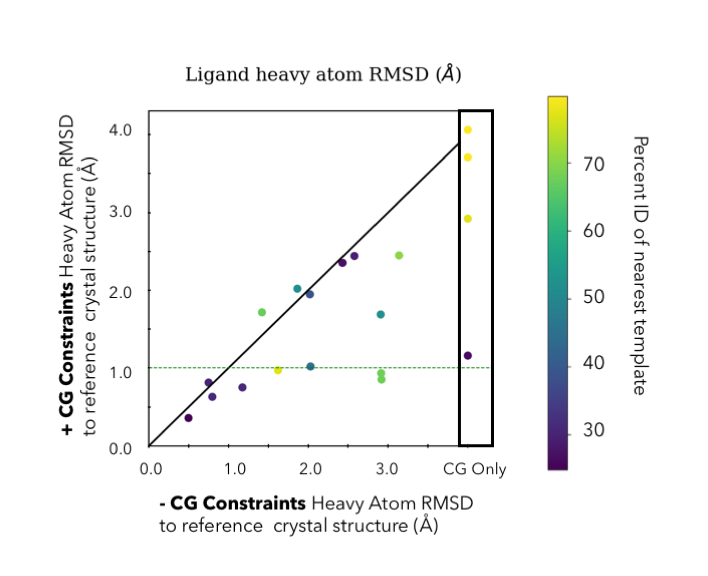

Supplement: S3 Fig — Results for docking by selecting the lowest single structure based on energy. One extreme point is not shown. (PNG) [file pone.0214126.s003.png]

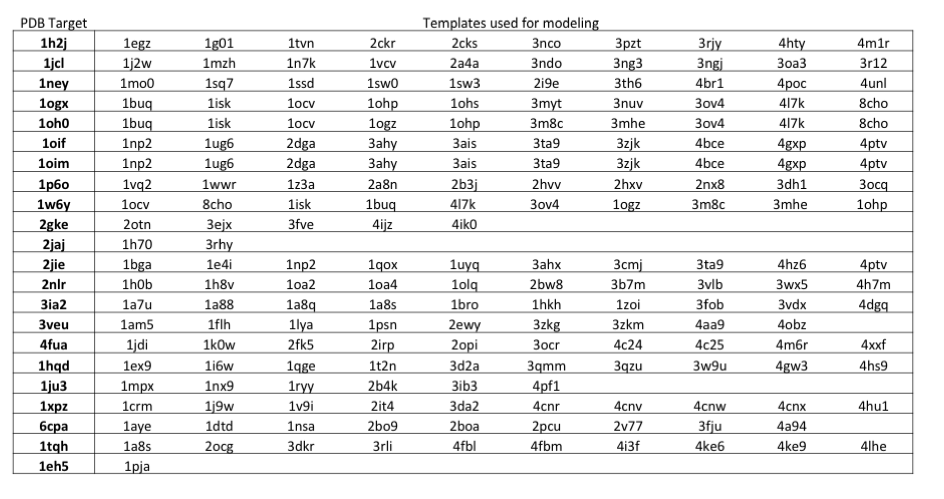

Supplement: S4 Fig — (PNG) [file pone.0214126.s004.png]
