# Supplementary material for: A new benchmark illustrates that integration of geometric constraints inferred from enzyme reaction chemistry can increase enzyme active site modeling accuracy
Source: PLoS One. 2019 Apr 4;14(4):e0214126. doi: 10.1371/journal.pone.0214126 (PMC6448891; doi:10.1371/journal.pone.0214126)
Supplement: S1 PDF — (PDF) [file pone.0214126.s005.pdf]

5 Benchmark Docking Results

For each target in the benchmark set, the crystal structure is shown in grey. The CG constraint models are shown in green, the models without CG constraints shown in red and the docking model (docking the ligand into the crystal structure) is shown in blue.

5.1 1h2j

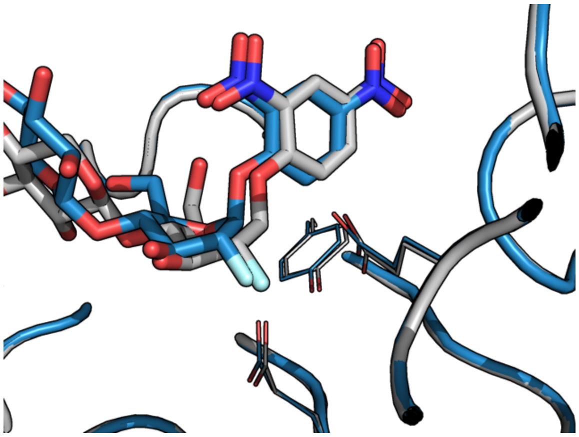

(a) Crystal structure docking recovery (blue).

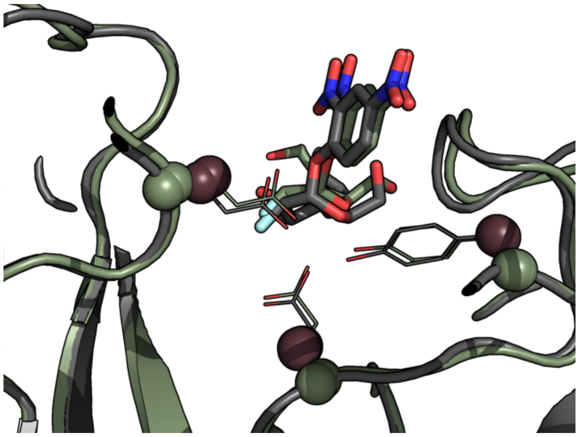

(b) **+ CG constraints** (green)

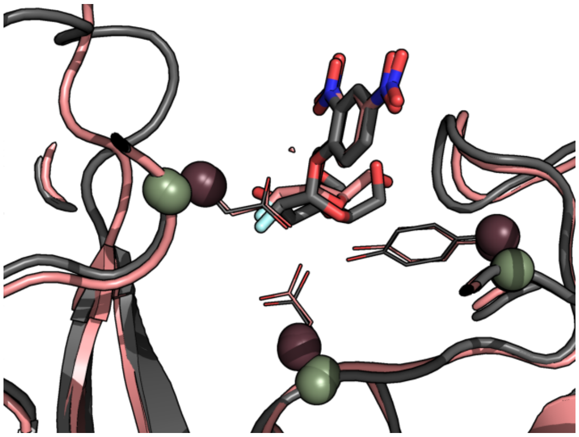

(c) **- CG constraints** (red)

$+CG$ LigRMSD : 1.3Å Active RMSD : 0.5Å

$-CG$ LigRMSD : 1.3Å Active RMSD : 1.2Å

5.2 1jcl

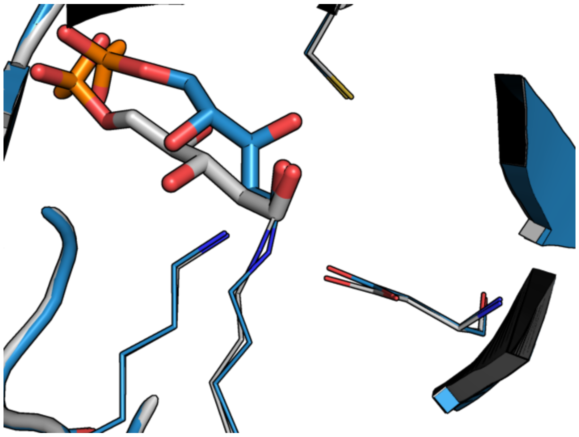

(a) Crystal structure docking recovery (blue).

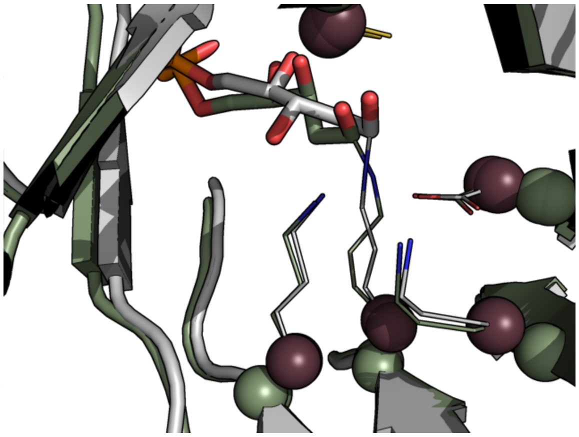

(b) + **CG constraints** (green)

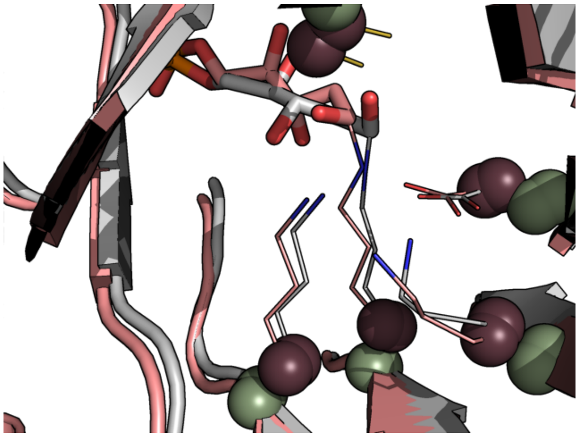

(c) **- CG constraints** (red)

$+CG$ LigRMSD : 2.3Å	Active RMSD : 1.2Å
$-CG$ LigRMSD : 2.3Å	Active RMSD : 0.8Å

5.3 1ney

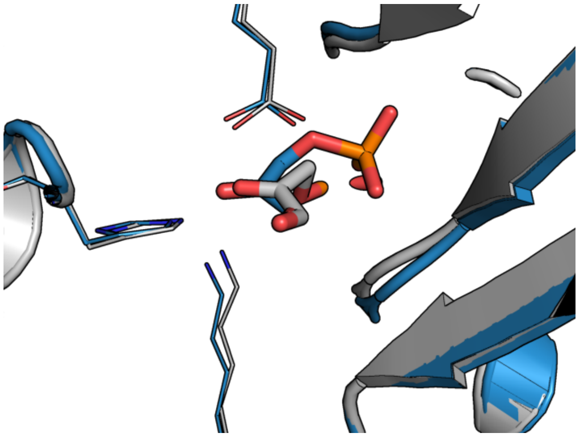

(a) Crystal structure docking recovery (blue).

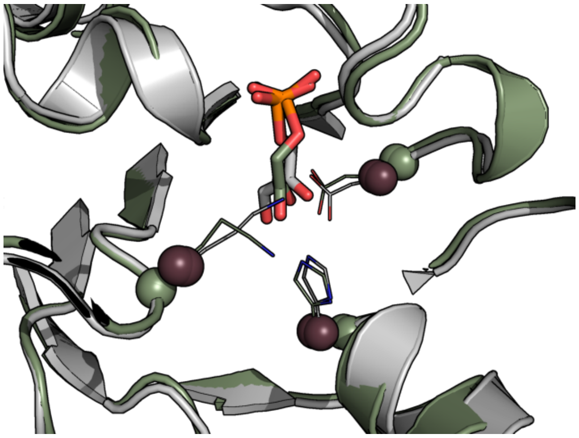

(b) **+ CG constraints** (green)

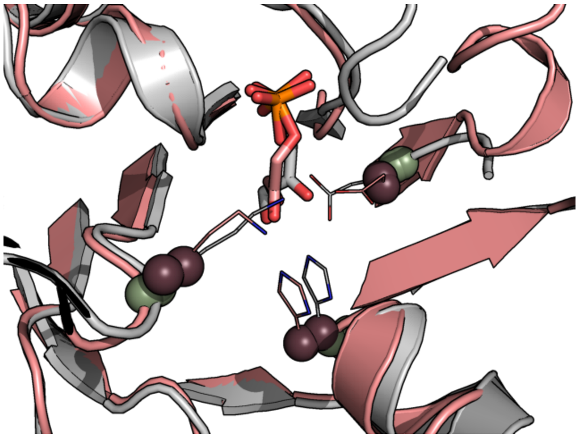

(c) **- CG constraints** (red)

$+CG$ LigRMSD : 2.0Å Active RMSD : 0.4Å
$-CG$ LigRMSD : 1.7Å Active RMSD : 2.1Å

5.4 1ogx

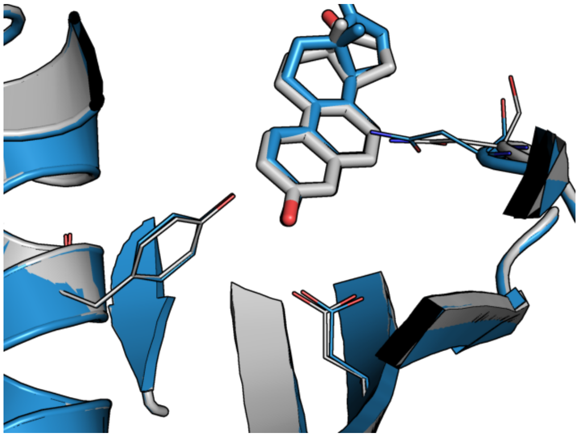

(a) Crystal structure docking recovery (blue).

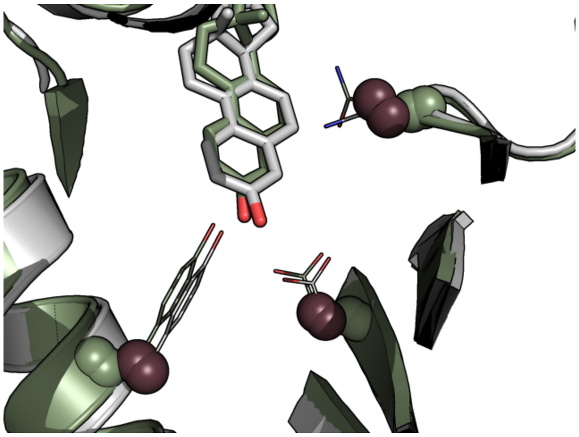

(b) **+ CG constraints** (green)

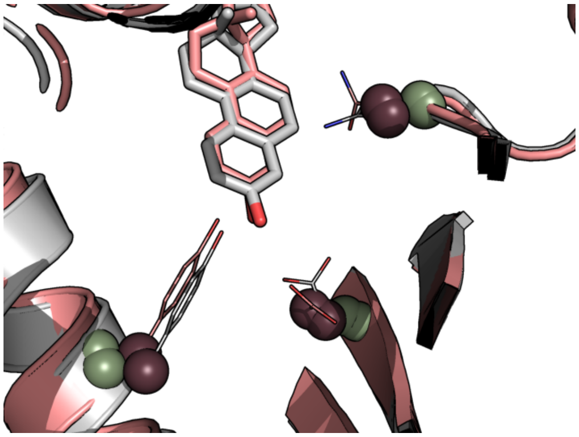

(c) **- CG constraints** (red)

$+CG$ LigRMSD : 0.8Å	Active RMSD : 0.8Å
$-CG$ LigRMSD : 0.7Å	Active RMSD : 1.1Å

5.5 1oh0

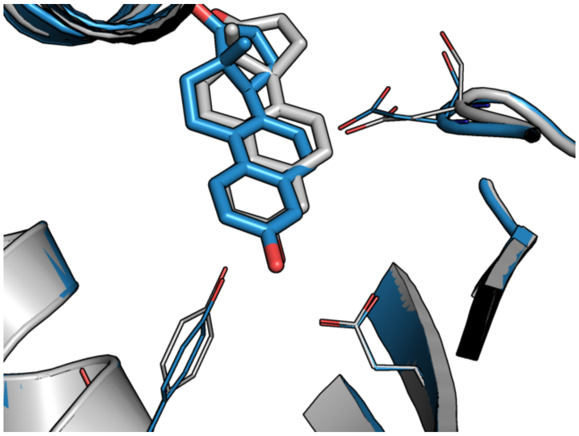

(a) Crystal structure docking recovery (blue).

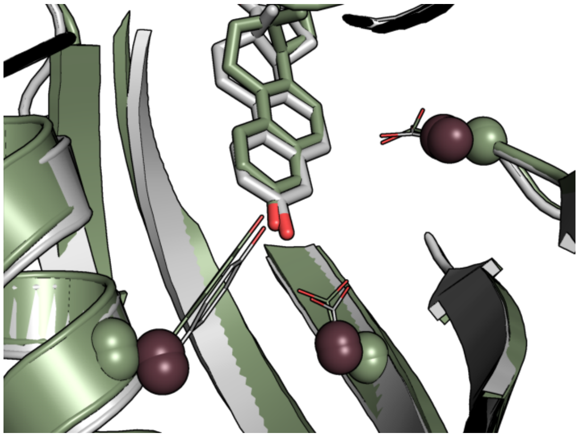

(b) + **CG constraints** (green)

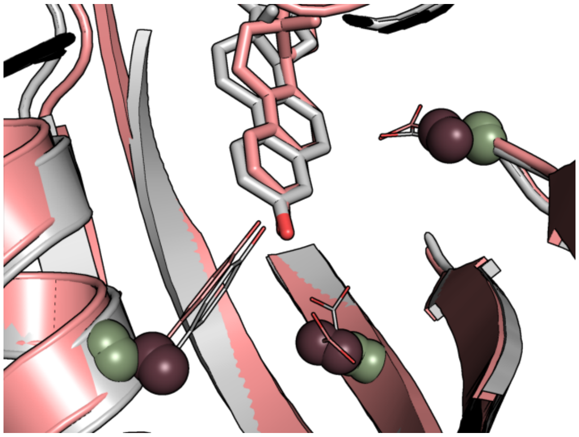

(c) **- CG constraints** (red)

$+CG$ LigRMSD : 0.6Å	Active RMSD : 0.8Å
$-CG$ LigRMSD : 0.8Å	Active RMSD : 1.4Å

5.6 1oif

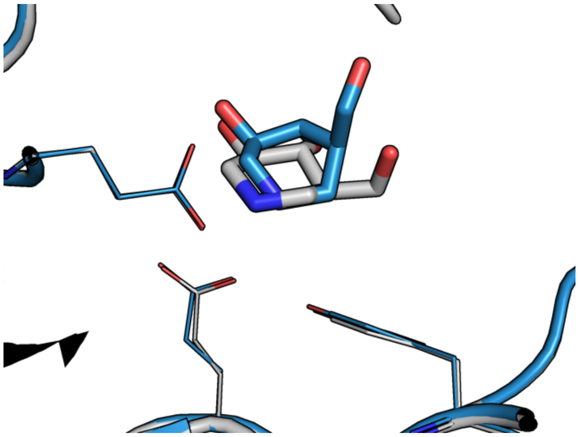

(a) Crystal structure docking recovery (blue).

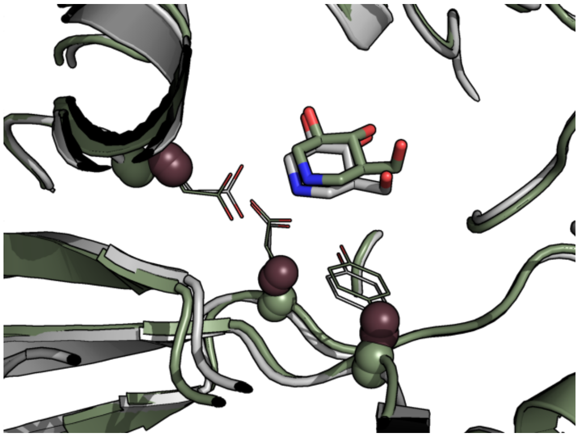

(b) + **CG constraints** (green)

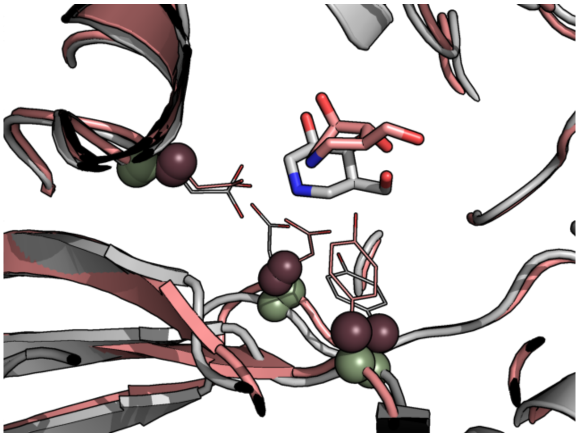

(c) **- CG constraints** (red)

$+CG$ LigRMSD : 0.9Å Active RMSD : 0.8Å
$-CG$ LigRMSD : 1.8Å Active RMSD : 0.5Å

5.7 1oim

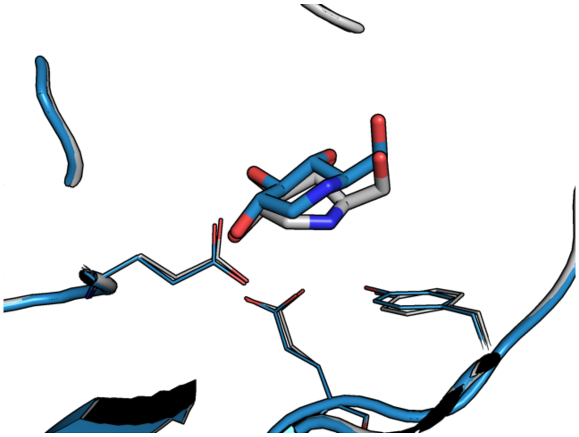

(a) Crystal structure docking recovery (blue).

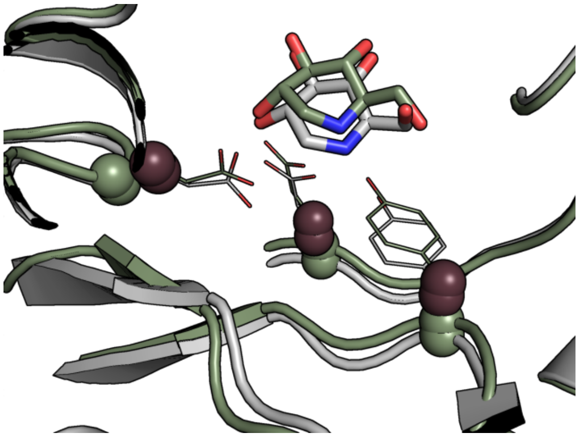

(b) + **CG constraints** (green)

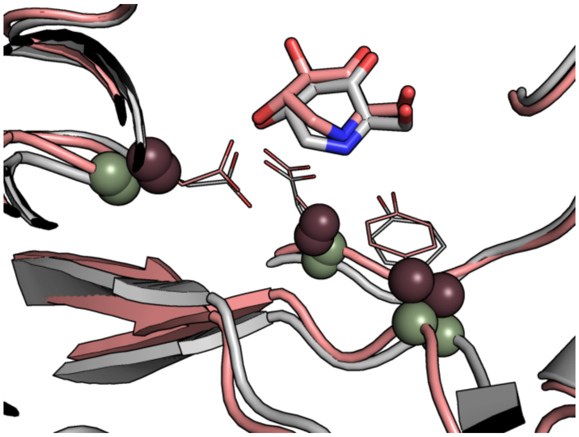

(c) **- CG constraints** (red)

$+CG$ LigRMSD : 0.8Å Active RMSD : 0.5Å
$-CG$ LigRMSD : 0.7Å Active RMSD : 0.8Å

5.8 1p6o

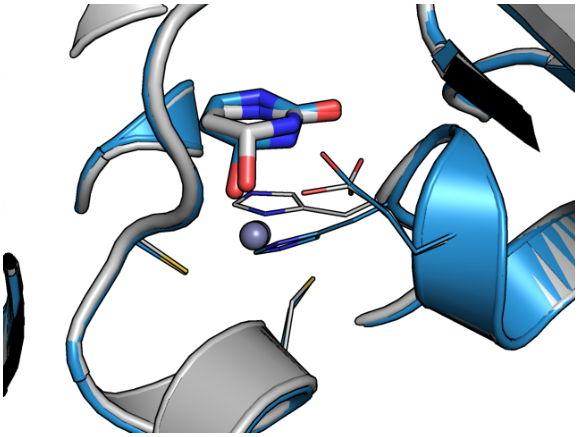

(a) Crystal structure docking recovery (blue).

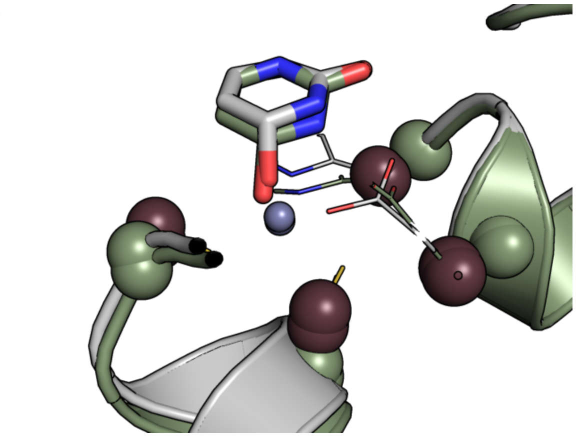

(b) **+ CG constraints** (green)

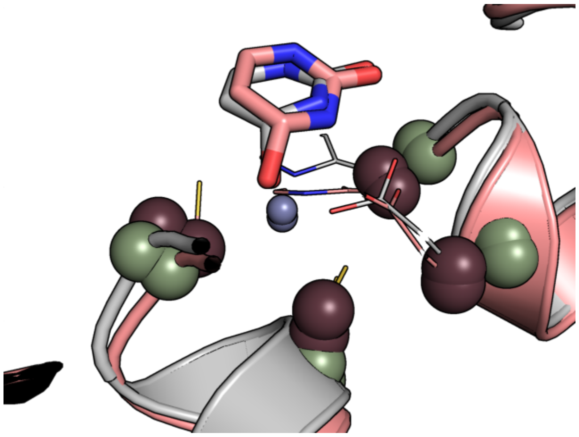

(c) **- CG constraints** (red)

$+CG$ LigRMSD : 0.3Å	Active RMSD : 6.1Å
$-CG$ LigRMSD : 0.5Å	Active RMSD : 7.3Å

5.9 1w6y

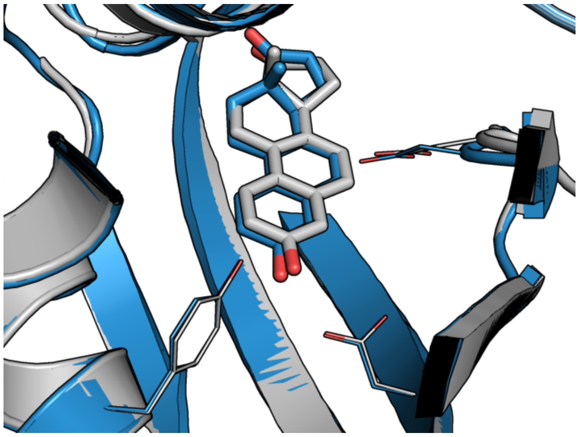

(a) Crystal structure docking recovery (blue).

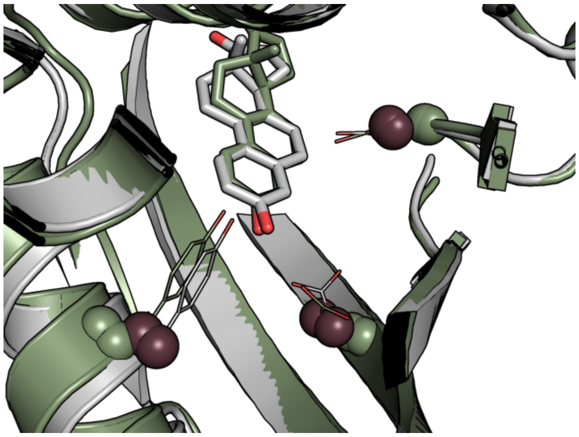

(b) **+ CG constraints** (green)

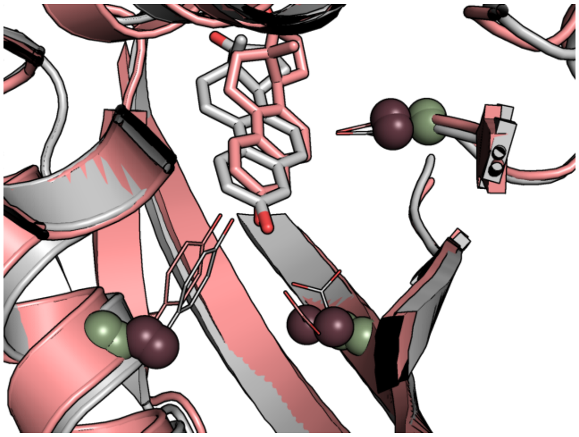

(c) **- CG constraints** (red)

$+CG$ LigRMSD : 0.8Å Active RMSD : 0.7Å
$-CG$ LigRMSD : 1.1Å Active RMSD : 1.0Å

5.10 2gke

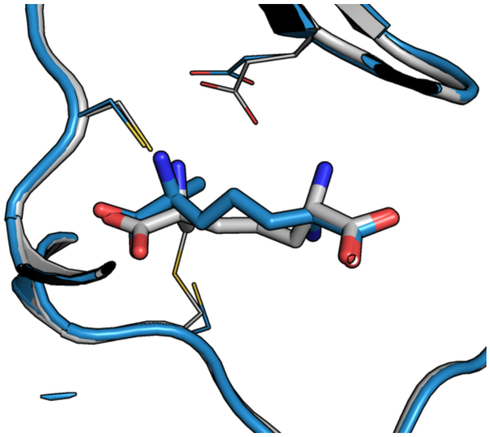

(a) Crystal structure docking recovery (blue).

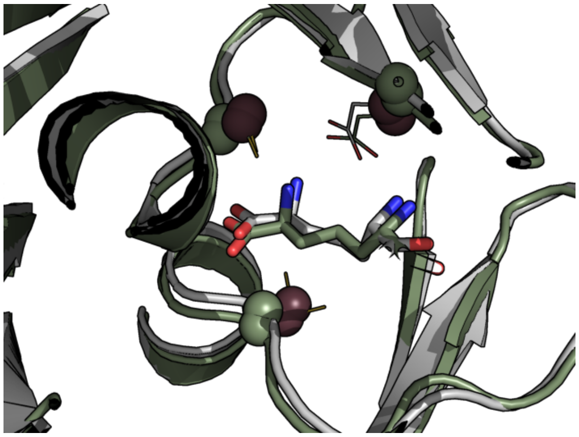

(b) **+ CG constraints** (green)

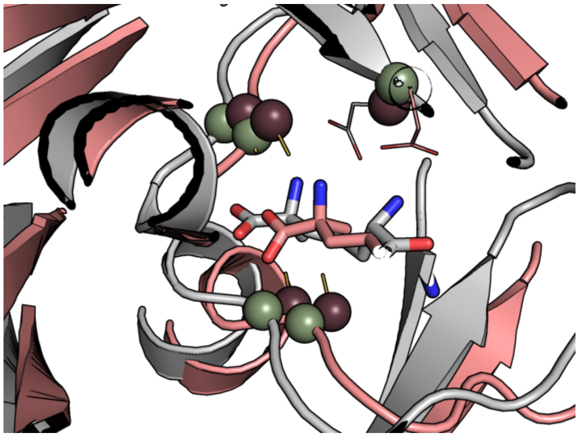

(c) **- CG constraints** (red)

$+CG$ LigRMSD : 1.0Å	Active RMSD : 0.6Å
$-CG$ LigRMSD : 1.6Å	Active RMSD : 2.7Å

5.11 2jaj

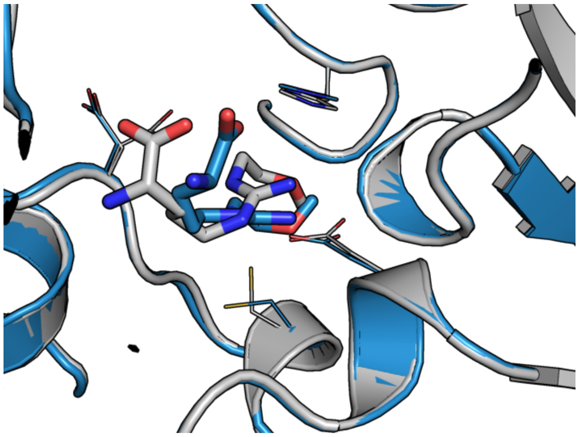

(a) Crystal structure docking recovery (blue).

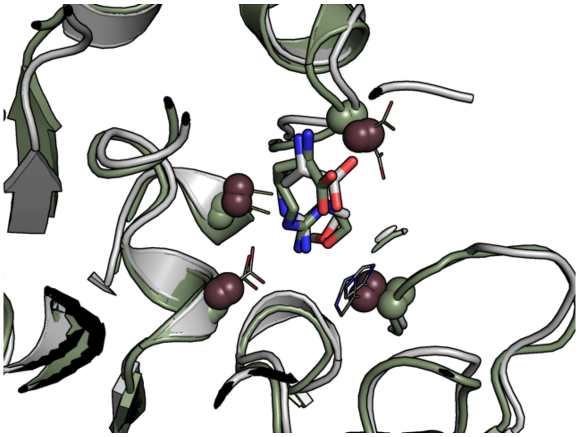

(b) + **CG constraints** (green)

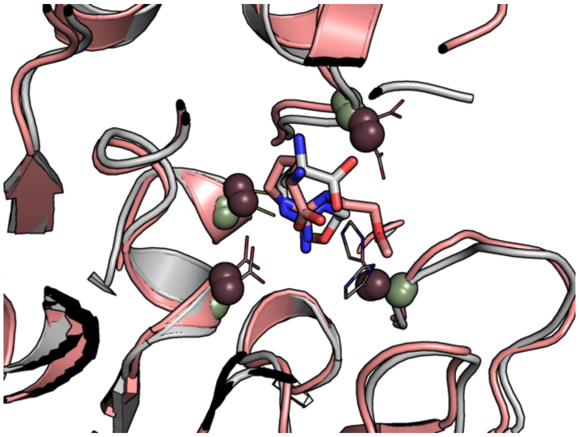

(c) **- CG constraints** (red)

$+CG$ LigRMSD : 0.9Å Active RMSD : 8.5Å*

$-CG$ LigRMSD : 2.2Å Active RMSD : 2.0Å

* Note: the low energy model for +CG has a tail that is flipped away from the active site. In the -CG model, the tail is flipped in. There are no CG constraints on the tail to keep it located near the active site, but the residues are included in the RMSD. This results in the active site having a large RMSD in the active site.

5.12 2jie

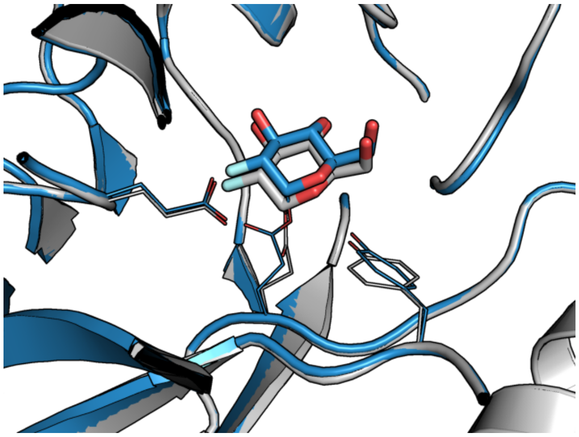

(a) Crystal structure docking recovery (blue).

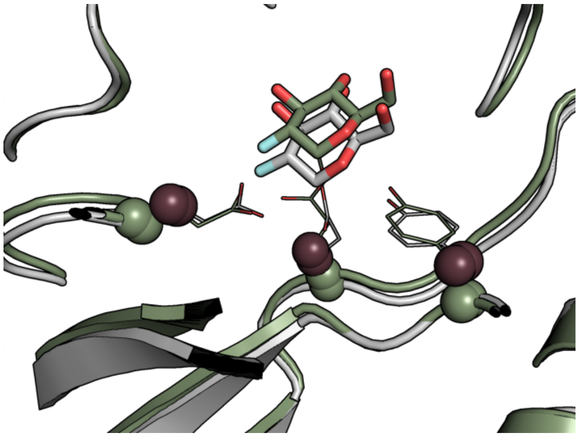

(b) **+ CG constraints** (green)

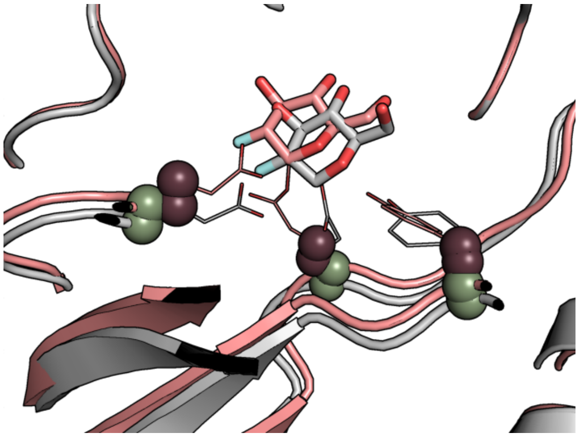

(c) **- CG constraints** (red)

$+CG$ LigRMSD : 0.9Å	Active RMSD : 0.7Å
$-CG$ LigRMSD : 1.3Å	Active RMSD : 0.9Å

5.13 2nlr

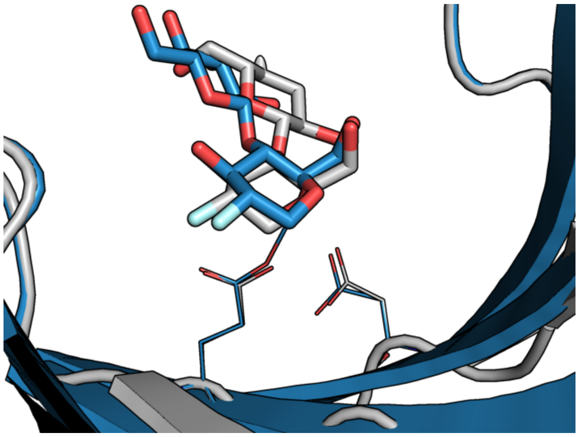

(a) Crystal structure docking recovery (blue).

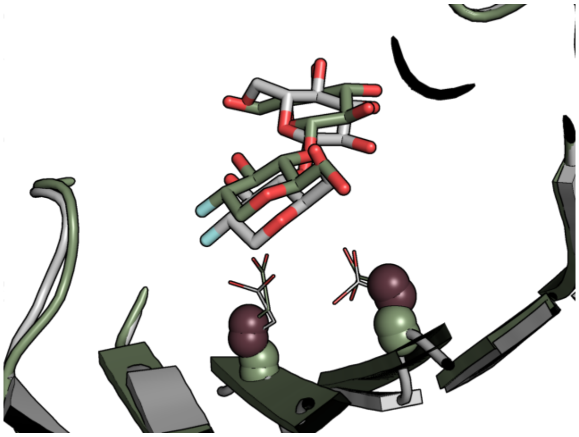

(b) + **CG constraints** (green)

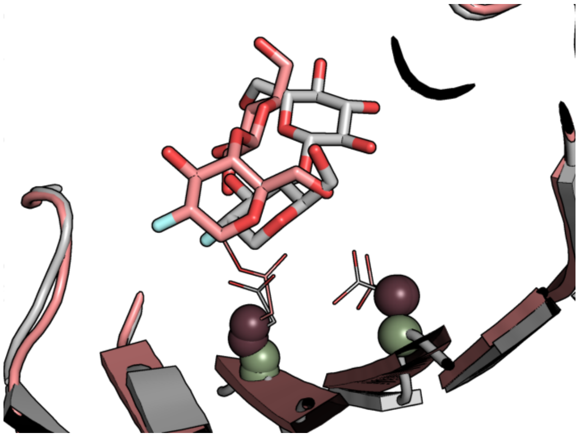

(c) - **CG constraints** (red)

$+CG$ LigRMSD : 0.9Å	Active RMSD : 0.7Å
$-CG$ LigRMSD : 2.7Å	Active RMSD : 0.8Å

5.14 3ia2

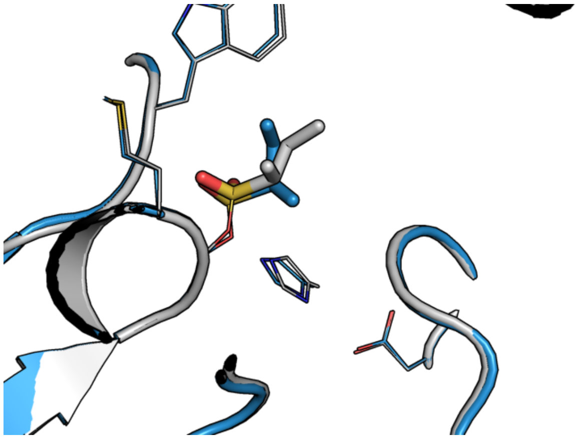

(a) Crystal structure docking recovery (blue).

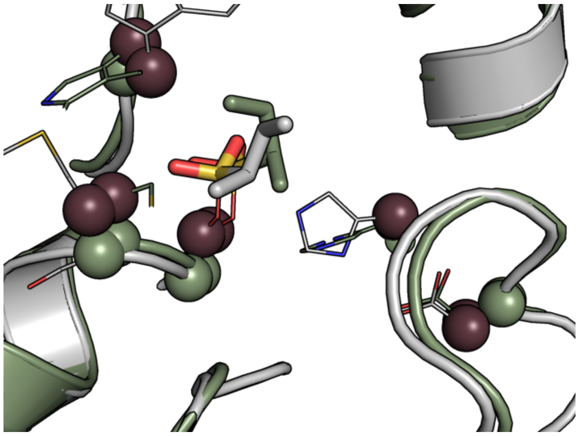

(b) + **CG constraints** (green)

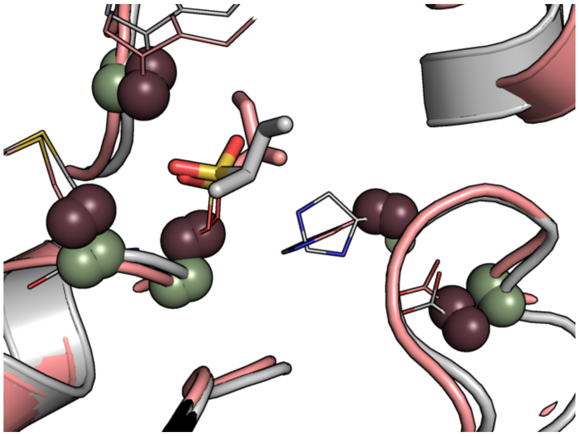

(c) **- CG constraints** (red)

$+CG$ LigRMSD : 1.7Å Active RMSD : 0.8Å

$-CG$ LigRMSD : 2.9Å Active RMSD : 0.9Å

5.15 3veu

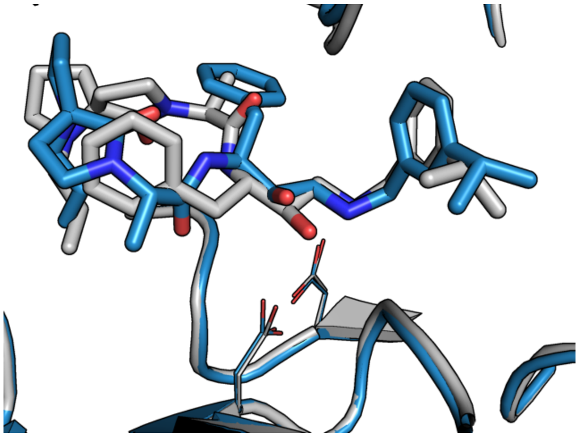

(a) Crystal structure docking recovery (blue).

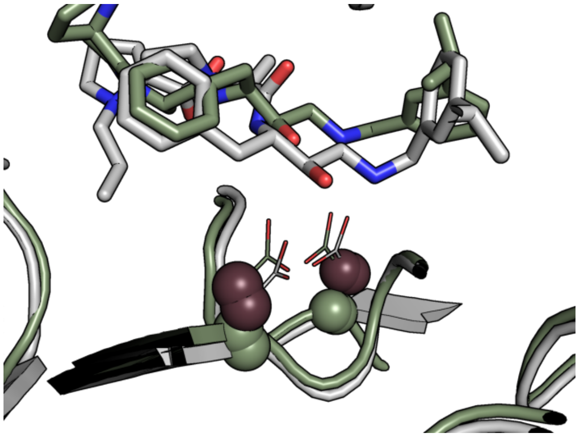

(b) + **CG constraints** (green)

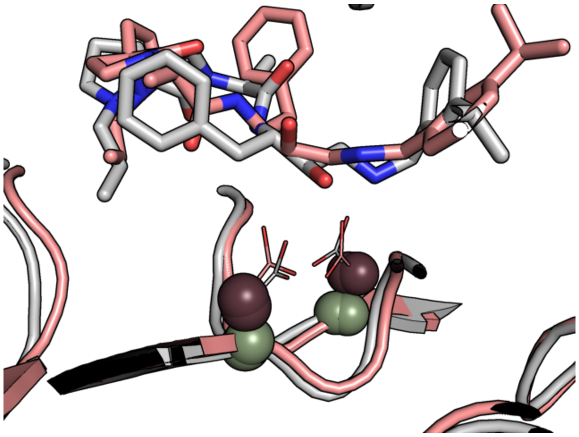

(c) **- CG constraints** (red)

$+CG$ LigRMSD : 3.6Å   Active RMSD : 1.1Å

$-CG$ LigRMSD : 3.0Å   Active RMSD : 1.2Å

5.16 4fua

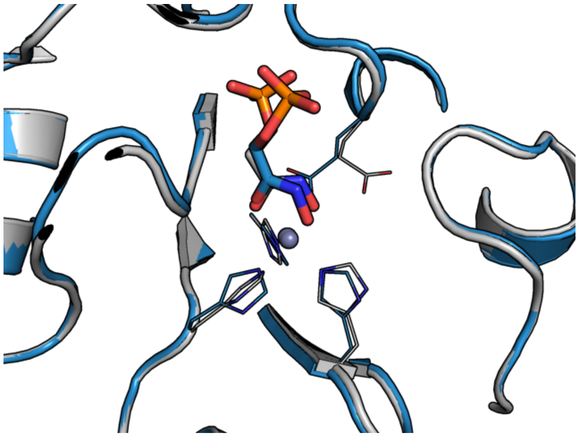

(a) Crystal structure docking recovery (blue).

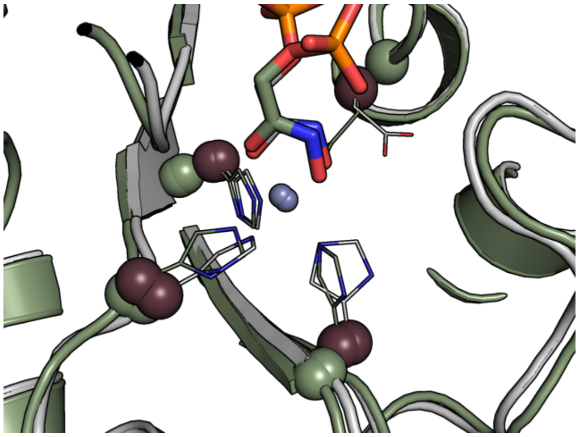

(b) + **CG constraints** (green)

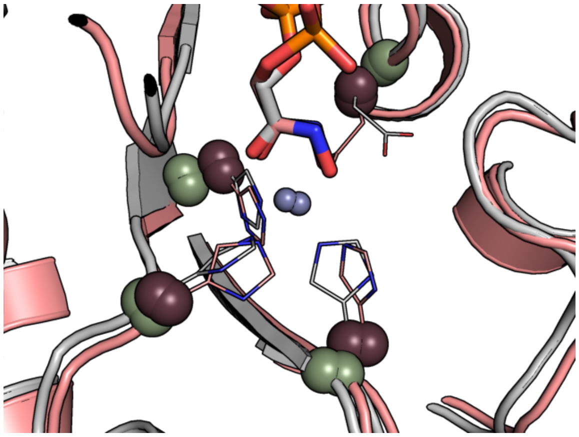

(c) **- CG constraints** (red)

$+CG$ LigRMSD : 1.9Å Active RMSD : 0.9Å

$-CG$ LigRMSD : 1.3Å Active RMSD : 0.9Å

Only solutions with addition of CG constraints found

In these structures, the models without CG constraints failed, in some particular way, to be consistent with known chemical interactions. Although they did not pass the filter to use in analysis, we have shown the lowest energy model, based on the same criteria, without removing models which fail the constraint energy filter. For example, the histidine residues in 6cpa fail to be able to all simultaneously coordinate the metal which is required for catalysis.

5.17 1hqd

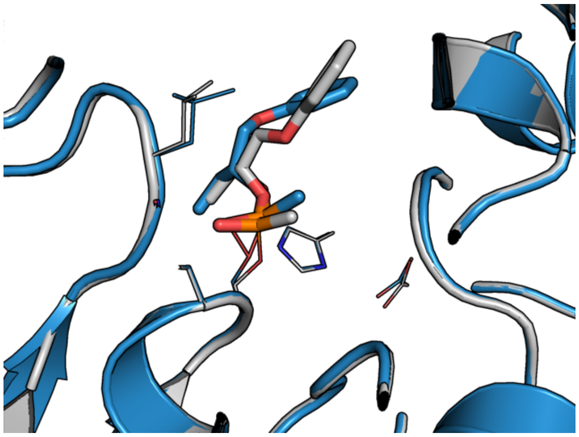

(a) Crystal structure docking recovery (blue).

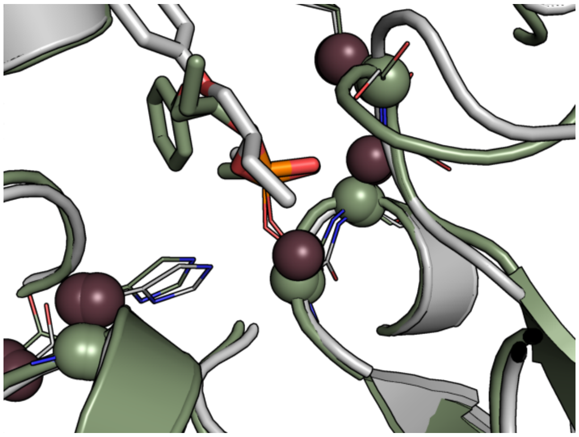

(b) + **CG constraints** (green)

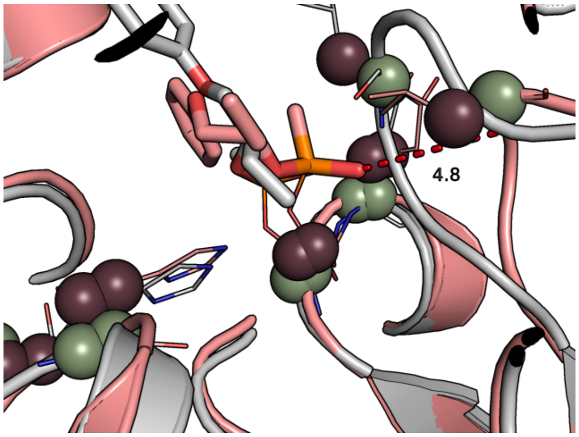

(c) **- CG constraints** (red)

$+CG$ LigRMSD : 2.9Å   Active RMSD : 1.2Å

$-CG$ Fails to create correct oxyanion hole distances

5.18 1ju3

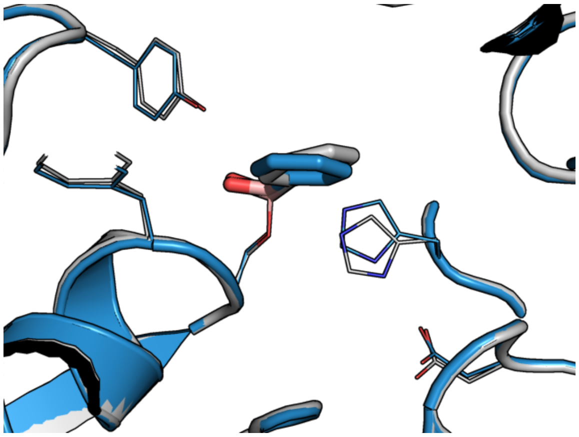

(a) Crystal structure docking recovery (blue).

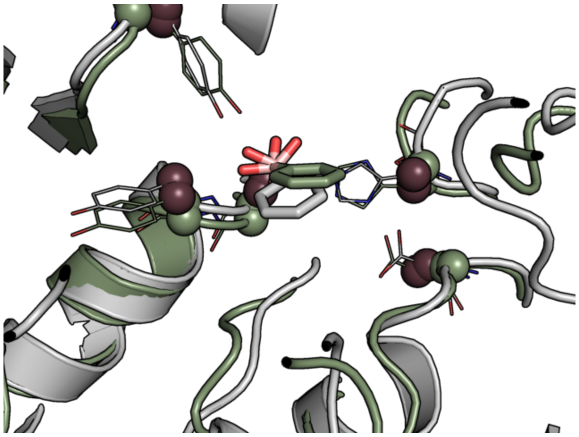

(b) + **CG constraints** (green)

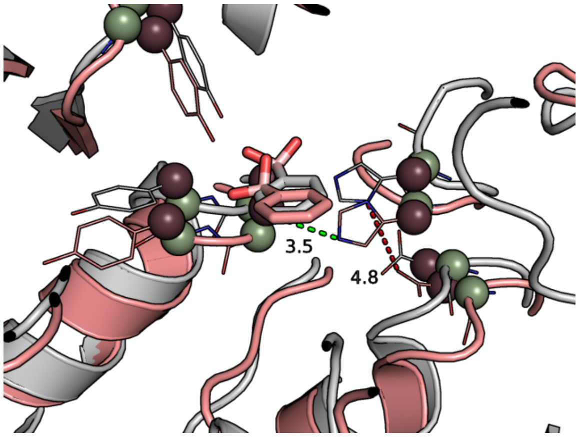

(c) **- CG constraints** (red)

$+CG$ LigRMSD : 1.2Å Active RMSD : 3.6Å

$-CG$ Fails to correctly set the HIS-ASP hydrogen bond network

5.19 1xpz

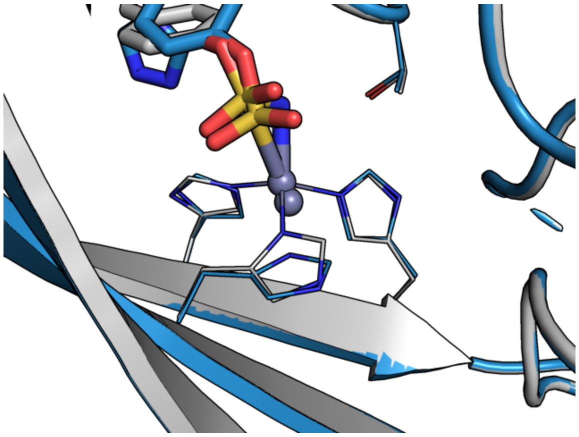

(a) Crystal structure docking recovery (blue).

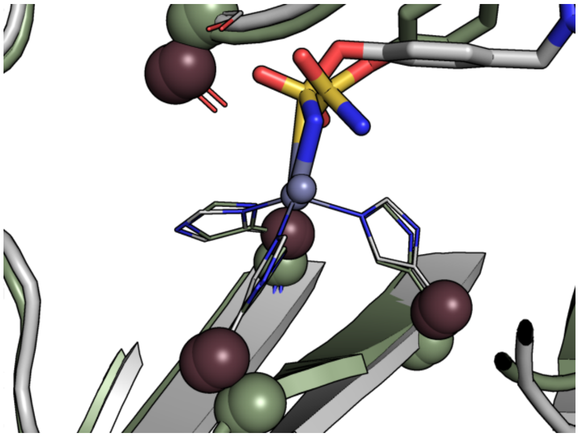

(b) **+ CG constraints** (green)

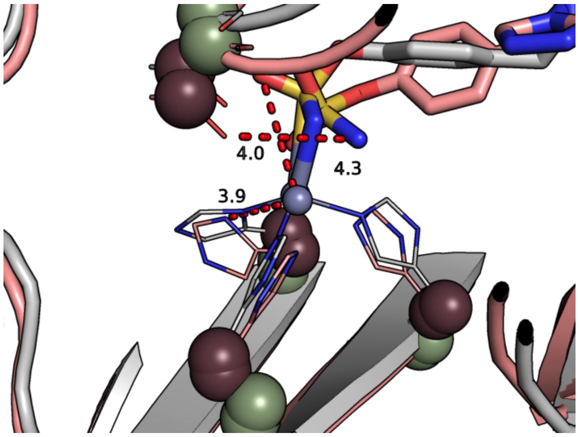

(c) **- CG constraints** (red)

+*CG* LigRMSD : 2.5Å Active RMSD : 0.8Å
CG Fails to meet a number of constraints

5.20 6cpa

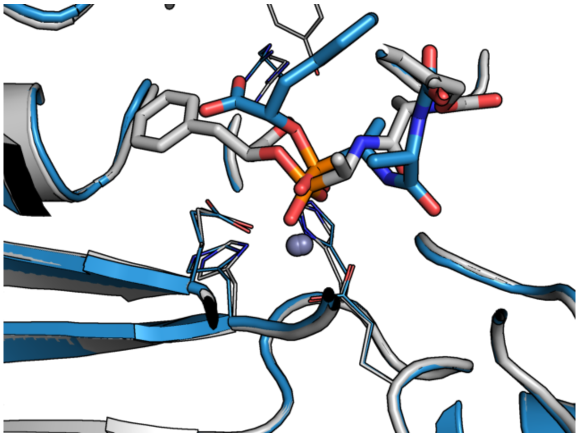

(a) Crystal structure docking recovery (blue).

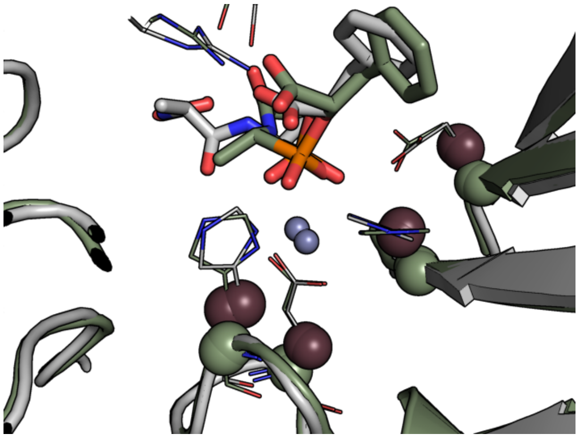

(b) **+ CG constraints** (green)

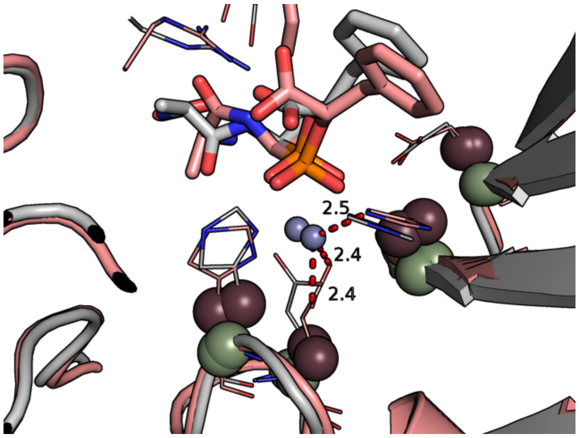

(c) **- CG constraints** (red)

$+CG$ LigRMSD : 2.8Å Active RMSD : 0.4Å
$-CG$ Fails to accurately bind the metal

No solutions found

5.21 1tqh

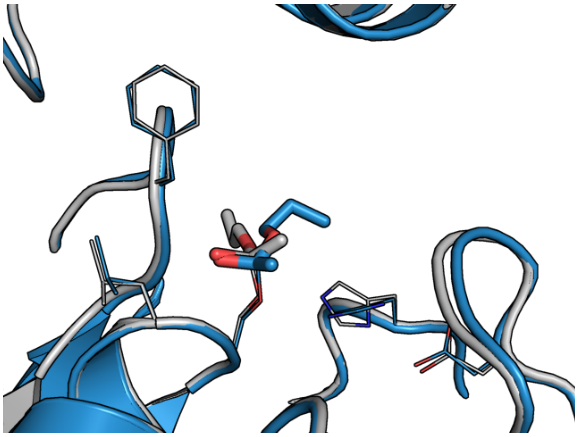

(a) Crystal structure docking recovery (blue).

5.22 1eh5

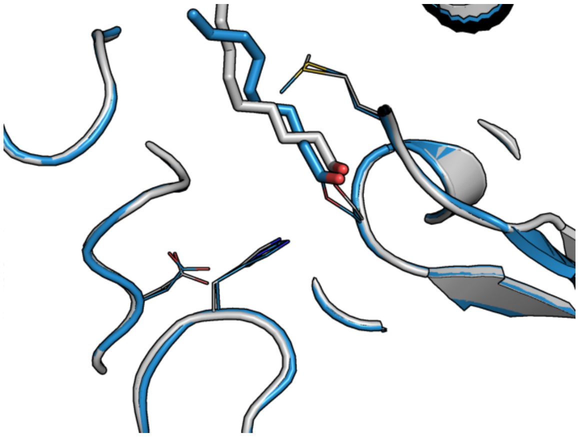

(a) Crystal structure docking recovery (blue).
