# Supplementary material for: A new benchmark illustrates that integration of geometric constraints inferred from enzyme reaction chemistry can increase enzyme active site modeling accuracy
Source: PLoS One. 2019 Apr 4;14(4):e0214126. doi: 10.1371/journal.pone.0214126 (PMC6448891; doi:10.1371/journal.pone.0214126)
Supplement: S2 PDF — (PDF) [file pone.0214126.s006.pdf]

1 Example of harmonic distance constraints

Constraints in Rosetta are implemented as follows:

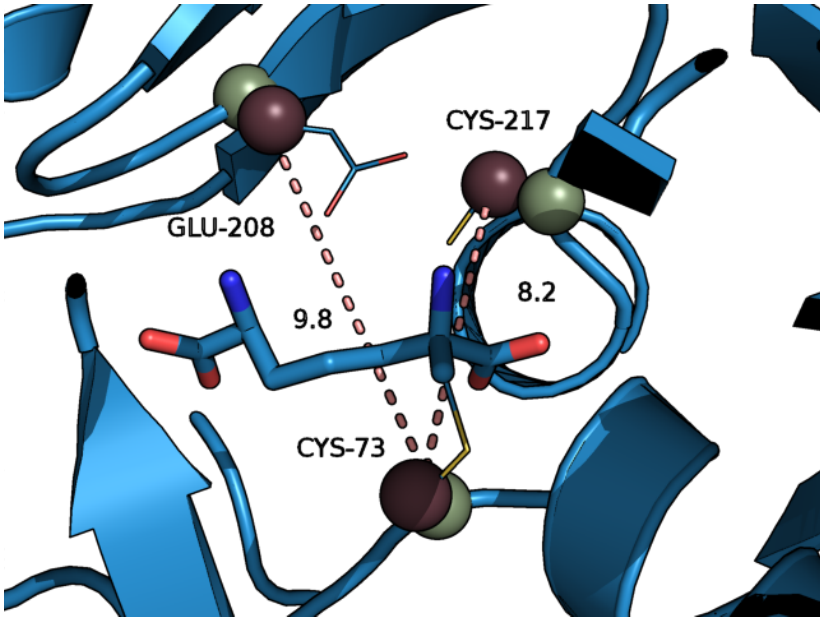

Figure 1: Example of measurements between catalytic residue C_α and C_β atoms. Shown are the C_β - C_β between CYS-73 to GLU-208 and CYS-217 (lines 1 and 2 below)

```
AtomPair CB 73 CB 208 SCALARWEIGHTEDFUNC 1000 HARMONIC 9.43 0.5
AtomPair CB 73 CB 217 SCALARWEIGHTEDFUNC 1000 HARMONIC 8.23 0.5
AtomPair CA 73 CA 208 SCALARWEIGHTEDFUNC 1000 HARMONIC 10.79 0.5
AtomPair CA 73 CB 208 SCALARWEIGHTEDFUNC 1000 HARMONIC 9.78 0.5
AtomPair CB 73 CA 208 SCALARWEIGHTEDFUNC 1000 HARMONIC 10.57 0.5
AtomPair CA 73 CA 217 SCALARWEIGHTEDFUNC 1000 HARMONIC 7.78 0.5
AtomPair CA 73 CB 217 SCALARWEIGHTEDFUNC 1000 HARMONIC 7.99 0.5
AtomPair CB 73 CA 217 SCALARWEIGHTEDFUNC 1000 HARMONIC 8.03 0.5
AtomPair CA 208 CA 217 SCALARWEIGHTEDFUNC 1000 HARMONIC 7.76 0.5
AtomPair CB 208 CB 217 SCALARWEIGHTEDFUNC 1000 HARMONIC 5.72 0.5
AtomPair CA 208 CB 217 SCALARWEIGHTEDFUNC 1000 HARMONIC 6.37 0.5
AtomPair CB 208 CA 217 SCALARWEIGHTEDFUNC 1000 HARMONIC 6.96 0.5
```
