# Supplementary material for: A new benchmark illustrates that integration of geometric constraints inferred from enzyme reaction chemistry can increase enzyme active site modeling accuracy
Source: PLoS One. 2019 Apr 4;14(4):e0214126. doi: 10.1371/journal.pone.0214126 (PMC6448891; doi:10.1371/journal.pone.0214126)
Supplement: S3 PDF — (PDF) [file pone.0214126.s007.pdf]

6 Modeling Details and Description of Files

6.1 Modeling Details

The following version of Rosetta was used to perform the work: 17be250fab3b65d60d806025d7219a5373754924

The benchmark files can be found (excluding the fragment files) at

https://www.github.com/sjbertolani/benchmark-lite

Description of files

6.2 Description of Files

Benchmark-

–Crystal Dock – target name All files included to run docking of ligand into the crystal structure using the ligand and enzyme design constraints. Execute run.sh on your cluster environment

–docking – target name These folders contain the ligand parameter files, the mol2 files from which they were derived, the enzyme design constraint files and the conformation libraries for the ligands. In some cases, there are utility scripts to modify the conformational libraries or params files.

–homologoussequences – target name These folders contain the results from searching for templates for each target sequence. Including the initial search results (pdb.json, pdb.gz, pdb, pdb.clean), initial alignments and alignments after removing close homologues (greater than 80% PID).

–ligandmaps –target name These files map the corresponding ligand atoms from the Rosetta parameter files atom name to the crystal structure ligand name (in cleaned up PDB files in pdbstructures).

–pdbstructures These files contain the original PDB files, cleaned up PDB files in which the waters, other chains, other organic molecules have been removed. The PDB files were also required to be renumbered for analysis, as well as some residues (SUI for example) needed to be removed. In some cases, the target ligand was denoted as multiple residues (ex. NAG-NAG) but were merged for analysis. The files ending in pbd.clean.renum have numbering that matches the output lowenergy structures to aid in analysis.

–lowenergy This folder contains the final output structures from which all the docking and active site data was calculated from.

–sequences These files contain the sequence as downloaded from the PDB. These contain the His tag. These sequences are modified and shortened in some cases due to lack of coverage by templates.

–structuralinfo These files (ending in templatescatres) include the mapping of the target catalytic residues to the equivalent catalytic residues for the template. Example: The line "1h2j E139 Y202 E228" in 1h2j identifies the catalytic residues as residues E139, Y202, and E228 in the crystal structure. In that same file, the line "3nco E144B Y205B E260B" denotes that in the template 3nco, the catalytic residues that is equivalent to Y202 is Y205 on chain B. These files are used to calculate the data for figure 1 in the main text. The other 2 files contain the raw data for the RMSD calculations show in figure 1 in the main text.

–templates –target name These files contain the template pdb structures, the threaded template pdb structures (template.pdb.pdb), the alignment of the target sequence to the templates (alignment.grishin, alignment.grishin.trim), a trimmed sequnece based on the template coverage (targetname.trim) , the upweighted constraint files which include both the evolutionary constraints (from James Thompsons work/ Robetta, alignment.grishin.trim.dist_csts) and the addition of the HARMONIC C_α - C_α, C_α - C_β and C_β - C_β terms from this work. Template pdb structures ending in ".clean2" were prepped for alignment in informatics analysis by removing alternate chains.

–models/ –target name –model Includes fragment files (3 and 9mer from benchmarking mode) trimmed sequence input for homology modeling Evolutionary constraints - alignment.grishin.dist_csts.bb_sc.CA homology modeling xml protocol - hybridize.xml flags and weights header file used for docking submission command - epiph.sh

–cgadded same as the model, but instead of the alignment.grishin.dist_csts.bb_sc.CA, has a file which the upweighted CG constraints, combined with the evolutionary constraints

–dockingfiles submission script example docking xml protocol -¿ the startfrom xyz coordinates need to be changed to the average xyz coordinates of the catalytic residues for the low energy model that is selected. header - contains the header that needs to be added to the low energy model before docking.

flags * also, the pdb of the ligand needs to be appended to the bottom of the low energy model before docking.

6.3 Additional Resources

github : https://github.com/sjbertolani/Calculation_of_Catalytic_Geometry_Constraints

This public repo contains example code to calculate the HARMONIC C_α-C_α, C_α-C_β and C_β-C_β terms, given the residue numbers of the catalytic residues

and a solved PDB crystal structure.
